# *white panicle*2 encoding thioredoxin *z*, regulates plastid RNA editing by interacting with multiple organellar RNA editing factors in rice

**DOI:** 10.1111/nph.17047

**Published:** 2020-12-19

**Authors:** Yunlong Wang, Yihua Wang, Yulong Ren, Erchao Duan, Xiaopin Zhu, Yuanyuan Hao, Jianping Zhu, Rongbo Chen, Jie Lei, Xuan Teng, Yuanyan Zhang, Di Wang, Xin Zhang, Xiuping Guo, Ling Jiang, Shijia Liu, Yunlu Tian, Xi Liu, Liangming Chen, Haiyang Wang, Jianmin Wan

**Affiliations:** ^1^ State Key Laboratory for Crop Genetics and Germplasm Enhancement Jiangsu Plant Gene Engineering Research Center Nanjing Agricultural University Nanjing 210095 China; ^2^ National Key Facility for Crop Resources and Genetic Improvement Institute of Crop Sciences Chinese Academy of Agricultural Sciences Beijing 100081 China

**Keywords:** MORFs, *Oryza sativa*, regulation, RNA editing, TRX, *WP2*

## Abstract

- Thioredoxins (TRXs) occur in plant chloroplasts as complex disulphide oxidoreductases. Although many biological processes are regulated by thioredoxins, the regulatory mechanism of chloroplast TRXs are largely unknown.
- Here we report a rice *white panicle2* mutant caused by a mutation in the thioredoxin z gene, an orthologue of AtTRX z in Arabidopsis. *white panicle2* (*wp2*) seedlings exhibited a high‐temperature‐sensitive albinic phenotype.
- We found that plastid multiple organellar RNA editing factors (MORFs) were the regulatory targets of thioredoxin z. We showed that OsTRX z protein physically interacts with OsMORFs in a redox‐dependent manner and that the redox state of a conserved cysteine in the MORF box is essential for MORF–MORF interactions. *wp2* and OsTRX z knockout lines show reduced editing efficiencies in many plastidial‐encoded genes especially under high‐temperature conditions. An Arabidopsis *trx z* mutant also exhibited significantly reduced chloroplast RNA editing.
- Our combined results suggest that thioredoxin z regulates chloroplast RNA editing in plants by controlling the redox state of MORFs.

## Introduction

Thioredoxins (TRXs) are small disulphide oxidoreductases essential for redox regulation of protein functions in all living organisms. All TRXs contain a redox‐active disulfide bridge with conserved CXXC (where X indicates a variable residue) thioredoxin‐box motif, which is involved in thiol/disulphide exchanges with target proteins to directly modulate the activity of the target proteins (Buchanan & Balmer, 2005). The *Arabidopsis thaliana* genome codes for more than 20 typical TRX proteins and about 30 TRX‐like proteins (Meyer *et al*., 2005, 2012). These TRX proteins belong to seven major groups: *f, m*, *h*, *o*, *x*, *y* and *z*, and are located in various subcellular compartments including chloroplasts, mitochondria and the cytosol. Earlier studies have shown that TRX proteins regulate many biological processes (Balmer *et al*., 2003, 2004, 2006; Wong *et al.*, 2004; Marchand *et al.*, 2006). The chloroplast TRX system is particular complex. Here, 10 TRXs in Arabidopsis are targeted to the chloroplast include two *f*‐type, four *m*‐type, one *x*‐type, two *y*‐type and one *z*‐type (Meyer *et al*., 2005, 2012; Arsova *et al.*, 2010). The *m*‐type and *f*‐type TRXs have mainly been implicated in redox regulation of photosynthetic carbon assimilation (Issakidis‐Bourguet *et al.*, 2001; Meyer *et al.*, 2005), whereas *x*‐type and *y*‐type TRXs are involved in protecting the plastid against oxidative damage (Collin *et al.*, 2003; Collin, 2004). Among the many typical chloroplast TRXs, TRX z exhibits some unique properties that are distinct from other plastid TRXs, such as a much lower expression level and an ability to be reduced by other plastid TRXs (Bohrer *et al.*, 2012). AtTRX z was initially identified in Arabidopsis as a component of plastid transcriptionally active chromosome (pTAC) (Pfalz *et al.*, 2006). Further studies have shown that TRX z functions as a component of plastid‐encoded RNA polymerase (PEP) and interacts with fructokinase‐like proteins FLN1 and FLN2 that were also identified as components of pTACs (Arsova *et al.*, 2010; Lv *et al.*, 2017; He *et al.*, 2018). TRX z also was reported to interact with other proteins, including the CHLI subunit of Mg‐chelatase, a putative plastidic oxidoreductase TSV, and Arabidopsis PLASTID REDOX INSENSITIVE2 (PRIN2) (Zhang *et al.*, 2015; Sun *et al.*, 2017; Diaz *et al.*, 2018). However, the molecular function and regulation of the TRX z protein remain largely unexplored.

RNA editing in flowering plants converts cytidine nucleotides (C) to uridine (T) in transcripts of both plastidial and mitochondrial genes in a highly specific manner (Castandet & Araya, 2011). In Arabidopsis, there are more than 500 RNA editing sites in mitochondria and 34 in plastids (Chateigner‐Boutin & Small, 2007; Bentolila *et al.*, 2008). RNA editing usually generates a more conserved amino acid residue relative to that in homologous proteins from other organisms (Gualberto *et al.*, 1989). Thus, RNA editing is thought to be a correction mechanism for T to C mutations that have arisen in plastids and mitochondria (Chateigner‐Boutin & Small, 2010; Fujii & Small, 2011). Recent studies have shown that RNA editing is affected by external conditions, such as high temperature (Karcher & Bock, 1998; Nakajima & Mulligan, 2001; Karcher & Bock, 2002), fungal infection (Garcia‐Andrade *et al.*, 2013), salt stress (Rodrigues *et al.*, 2017), oxidative stress (Xiong *et al.*, 2017), antibiotics lincomycin (Lin) and norflurazon (NF) (Kakizaki *et al.*, 2012; Tseng *et al.*, 2013). However, the physiological significance of these effects and their underlying mechanisms are elusive.

In plants, RNA editing has been found for 30 yr (Covello & Gray, 1989). Recent molecular genetic studies have identified a number of factors that constitute the editosome complex required for organellar RNA editing. The PLS subfamily of pentatricopeptide repeat (PPR) proteins were identified as site‐specific recognition factors for C targets in plant organelle (Kotera *et al.*, 2005; Fujii & Small, 2011). In addition, some non‐PPR editing factors were identified more recently, including the multiple organellar RNA editing factors (MORFs)/RNA editing factor interacting proteins (Bentolila *et al.*, 2012; Takenaka *et al.*, 2012), the organelle RNA recognition motif‐containing proteins (Sun *et al.*, 2013; Shi *et al.*, 2016), organelle zinc‐finger 1 (OZ1) (Sun *et al.*, 2015) and protoporphyrinogen IX oxidase 1 (PPO1) (Zhang *et al.*, 2014). Despite these significant advances, the complete composition of the editing machinery and the regulatory mechanism of RNA editing remained largely unknown.

In this study, we characterised a rice *white panicle2* mutant (*wp2*) that exhibits an albinic phenotype at high temperature. Map‐based cloning showed that *WP2* encodes an OsTRX z protein. We show that OsTRX z interacts with OsMORFs both *in vivo* and *in vitro* and that OsTRX z–OsMORFs and OsMORF–OsMORF interactions are redox dependent. *Ostrx z* knockout lines exhibited inefficient chloroplast RNA editing especially at higher temperatures. An Arabidopsis *trx z* mutant also showed inefficient chloroplast RNA editing. Based on these results, we propose that TRX z participates in chloroplast RNA editing by regulating the redox status of MORFs.

## Materials and Methods

### Plant growth conditions

The *wp2* mutant was isolated from a T‐DNA enhancer‐tagged population of the subspecies *japonica* cultivar Nipponbare. Knockout plants of *OsTRX z* were obtained from L. He (He *et al.*, 2018). Rice plants were grown in a paddy field at Nanjing, under natural conditions. For qRT‐PCR, immunoblotting, RNA editing analysis and transmission electron microscopy, seedlings of the wild‐type and *wp2* mutant were grown in climate chambers at 70% humidity, under a 14 h : 10 h, light : dark (300 µmol m^−2^ s^−1^) regime at 25°C or 35°C. To test the effect of temperature shift on *WP2* expression, wild‐type seedlings were initially grown at 25°C for 10 d and then transferred to another climate chamber at 35°C. Arabidopsis *Attrx z* (Salk_028162C) mutants were obtained from the SALK collection and confirmed by PCR‐based genotyping using primers listed in Supporting information Table S4. The Arabidopsis seeds were sown on Murashige and Skoog agar plates with 2% sucrose and grown under a 16 h : 8 h, light : dark (150 µmol m^−2^ s^−1^) regime at 22°C (50% humidity).

### Measurement of photosynthetic pigment and transmission electron microscopy analyses

For pigment extraction, 300 mg fresh plant tissues were extracted in 5 ml of 95% ethanol. Specific pigment contents were determined using SpectraMax M3 (Molecular Devices) according to the method descried by Arnon (Arnon, 1949). Tissues of wild‐type and *wp2* mutant for transmission electron microscopy, were fixed in 2.5% glutaraldehyde at 4°C for 16 h, rinsed, and incubated overnight in 1% O_s_O_4,_ stained with uranyl acetate, dehydrated in an ethanol series, and finally embedded in Spurr’s medium before ultrathin sectioning. Samples were stained again with uranyl acetate and observed under a Hitachi H‐7650 transmission electron microscope.

### Positional cloning and complementation

To map the *WP2* locus, *wp2* was crossed with rice cultivar IR36 (*indica*). Twenty F_2_ progenies with white panicles from the *wp2*/IR36 cross were used for the initial chromosome location. Then 2057 F_2_ plants with the recessive *wp2* phenotype were used for genetic mapping. The markers used for positional cloning are listed in Table S4. For complementation of the *wp2* mutant, the *LOC_Os08g29110* coding sequence was cloned into the pCAMBIA1300‐221‐3*FLAG binary vector. The construct was introduced into *Agrobacterium tumefaciens* (strain EHA105) and then transformed into *wp2* mutant calli. Hygromycin‐resistant calli were regenerated and seedlings were grown in a glasshouse.

### Subcellular localisation

The coding sequences of *OsTRX z*, *OsMORF2* (*LOC_Os04g51280*), *OsMORF8* (*LOC_Os09g33480*) and *OsMORF9* (*LOC_Os08g04450*) were amplified and fused to the N‐terminus of GFP under control of the double CaMV35S promoter in transient expression vector pAN580‐GFP, whereas *OsMORF8* was fused to the N‐terminus of mCherry under the control of the double CaMV35S promoter in the transient expression vector pAN583‐mCherry. The above constructs were separately or simultaneously transformed into protoplasts isolated from 10‐d‐old rice seedlings and incubated in darkness at 28°C for 16 h before examination. Mitochondria were dyed with 0.5 mM MitoTracker Orange CM‐H2TMRos (Invitrogen) for 30 min and washed before observation using a confocal laser scanning microscope (Leica SP8).

### Co‐expression analysis

The Rice FREND database was used for co‐expression analysis of *OsTRX z* and *OsFLN1* (http://ricefrend.dna.affrc.go.jp/single‐guide‐gene.html). The first 100 co‐expressing genes were used for Gene Ontology (GO) enrichment analysis.

### Protein extraction and immunoblot analysis

Total proteins were isolated from 10‐d‐old rice seedlings. The tissues were ground in liquid nitrogen and thawed in an equal volume of extraction buffer (50 mM Tris‐HCl, pH 7.5, 150 mM NaCl, 1 mM EDTA, 0.1% Triton X‐100, 0.5 (v/v) β‐mercaptoethanol, one tablet/50 ml protease inhibitor cocktail; Roche) for 30 min on ice. Cell debris was removed by centrifugation at 12 000 ***g*** for 15 min at 4°C. Total proteins were separated by SDS‐PAGE, transferred to polyvinylidene difluoride (PVDF) membrane (45 μm; Millipore), followed by immunoblotting with OsTRX z polyclonal antibodies (dilution 1 : 1000) raised against the peptide SPDQSKDALRTEC in rabbits. Antibodies were antigen affinity purified from the immune serum.

### Yeast‐two‐hybrid assays

Yeast‐two‐hybrid experiments were performed according to ‘Yeast protocols’ in the *Handbook and Matchmaker GAL4 Two‐hybrid System 2* manual (Clontech). Briefly, the respective combinations of GAL4 DNA binding domain and GAL4 activation domain fusions were cotransformed into the yeast strain AH109. The transformants were grown on SD/−Trp−Leu (DDO) and SD/−Trp−Leu−His−Ade (QDO) dropout plates, respectively.

### Bimolecular fluorescence complementation (BiFC) assay

The full‐length coding sequences of *OsTRX z* and *OsRbcS* were cloned into binary vector pSPYNE173, and the full‐length coding sequences of *OsMORF2*, *OsMORF8*, *OsMORF9* and *OsRbcS* were cloned into the binary vector pSPYCE(M). Plasmids containing N‐terminal and C‐terminal fusion of YFP were introduced into *Agrobacterium tumefaciens* (strain EHA105) and co‐infiltrated with p19 strain into 5‐wk‐old *N. benthamiana* leaves as described previously (Waadt *et al.*, 2008). BiFC‐induced fluorescence was detected using a confocal laser scanning microscope (Leica SP8) after 48 h of incubation at 22°C.

### RNA extraction and quantitative RT‐PCR analysis

Total RNA was isolated using the RNAprep Pure Plant kit (Tiangen). First‐strand cDNA was synthesised using PrimeScript™ II 1st Strand cDNA Synthesis kit (TaKaRa) and random hexamer primers (TaKaRa). Rice *UBIQUITIN* (*UBQ*) was used as an internal control. qRT‐PCR was performed using an ABI 7500 Real‐Time PCR system with SYBR Green Mix (Bio‐Rad) with three biological replicates. Primers used for qRT‐PCR are listed in Table S4.

### *In vitro* pull‐down assay

For *in vitro* pull‐down assays, OsTRX z, OsMORF2 and OsMORF9 were fused to the HIS‐tag (pET‐30a), and OsMORF8 was fused to the maltose binding protein (MBP)‐tag (pMAL‐c2X). The plasmids were transformed into *E. coli* (BL21). Supernatants of MBP‐OsMORF8 was first loaded onto the MBP‐Binding Resin (New England Biolabs, Ipswich, MA, USA) and washed with column buffer (20 mM Tris‐HCl pH 7.4, 200 mM NaCl, 1 mM EDTA). The supernatant of HIS‐OsTRX z/OsMORF2/OsMORF9 was loaded onto an MBP‐OsMORF8 binding resin column with or without DTT (20 mM) and then washed three times with column buffer, and resins were boiled with SDS‐PAGE sample buffer for 5 min. The eluted proteins were analysed by immunoblotting using antibody against HIS‐tag (Millipore; dilution 1 : 5000) and MBP‐tag (New England Biolabs; dilution 1 : 5000).

### Co‐immunoprecipitation assay

Full‐length coding sequences of *OsTRX z* and *OsMORF8* were cloned into the binary vector pCAMBIA1300‐221‐3*FLAG, and full‐length coding sequences of *OsMORF2*, *OsMORF8* and *OsMORF9* were cloned into the binary vector pCAMBIA1305.1‐GFP. All proteins were fused to the N‐terminus of their respective tags. For co‐immunoprecipitation experiments, agroinfiltrated tobacco leaves (48 hpi) were infiltrated with 1 mM dithio‐bis(succinimidyl propionate) (DSP) protein cross‐linker (Sigma) for 30 min. The reaction was terminated by incubating in 50 mM glycine for 30 min to quench the free DSP. The leaves were then ground in liquid nitrogen and lysed in an immunoprecipitation (IP) buffer (10 mM HEPES‐KOH, pH 7.5, 0.5% Nonidet P‐40, 2 mM EDTA, 150 mM NaCl, 10% glycerol, 5 mM NaF and one protease inhibitor cocktail tablet (Roche)). Samples were centrifuged at 12 000 ***g*** for 10 min at 4°C to remove cell debris. Protein extracts were incubated with 40 µl of protein A/G agarose beads (Roche) with rotation for 2 h at 4°C. After incubation, the beads were removed by centrifugation at 500 ***g*** for 2 min, and 20 µl supernatant was collected as the input fraction. The remaining supernatant was incubated with 20 µl anti‐Flag antibody M2 agarose beads (Sigma) for 2–3 h with rotation at 4°C. The beads were washed three times with 1 ml buffer, and the bound proteins were eluted by boiling the beads in SDS‐PAGE sample buffer for 5 min. For DTT treatment, 20 mM DTT was added to the IP buffer and the DSP infiltration step was omitted. The eluted proteins were analysed by immunoblotting using antibodies against Flag‐tag (Abgent; dilution 1 : 2000) and GFP‐tag (Genscript dilution 1 : 2000 and Abcam dilution 1 : 5000), respectively.

### RNA editing assay

Total RNA was isolated using the RNAprep Pure Plant kit (Tiangen), and first‐strand cDNA was synthesised using the PrimeScript II 1st Strand cDNA Synthesis Kit and random hexamer primers (TaKaRa). RNA editing efficiencies in rice and Arabidopsis were measured primarily by bulk sequencing of RT‐PCR products amplified with primers according to previously reported procedures (Robbins *et al.*, 2009; Zhang *et al.*, 2017). The RNA editing levels for each site were measured by the relative height of the peak of the nucleotide in sequence chromatograms and calculated by the height of ‘T’ divided by the sum of the height of ‘T’ and ‘C’. Four repeats were used for statistical analysis.

## Results

### Phenotypic characterisation of the *wp2* mutant

**Fig. 1:**
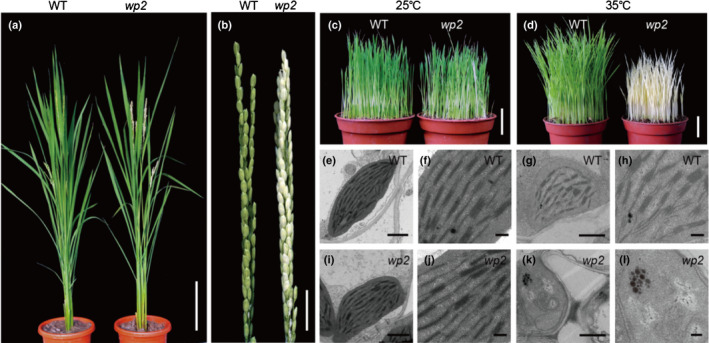
Phenotype of the rice *wp2* mutant. (a) Phenotype of wild‐type and *wp2* mutant at the heading stage. (b) Panicles of wild‐type and *wp2* mutant. (c, d) Phenotypes of 10‐d‐old wild‐type and *wp2* mutant seedlings grown at 25°C (c) and 35°C (d). (e–l) Chloroplast ultrastructure of wild‐type (e–h) and *wp2* (i–l) seedlings grown at 25°C (e, f, i, j) and 35°C (g, h, k, l). The fourth leaf of 10‐d‐old seedlings was used for transmission electron microscopic observation. Bars: (a) 15 cm; (b) 2 cm; (c, d) 3 cm; (e, g, i, k) 1 µm; (f, h, j, l) 200 nm.

The *wp2* mutant is a rice somaclonal mutant of the *japonica* variety Nipponbare that exhibits white panicles at the heading stage in the field (Fig. 1a,b). Transmission electron microscopy of panicles from the *wp2* mutant showed that chloroplast shape was abnormal and the thylakoids were severely disrupted (Fig. S1a,b). Seedlings of the *wp2* mutant were albinic when grown at 35°C, but were indistinguishable from wild‐type seedlings grown at 25°C (Fig. 1c,d). Pigment contents and chloroplast ultrastructure in *wp2* mutant and wild‐type seedlings were almost identical when grown at 25°C (Figs 1e,f,i,j, S1c). However, in *wp2* seedlings the pigment contents were significantly decreased at 35°C (Fig. S1d). Chloroplasts of *wp2* were abnormal in shape and contained no thylakoid membranes at 35°C (Fig. 1g,h,k,l). It is worth noting that temperatures around the heading date under normal paddy conditions are much higher than those at the seedling stage. This could be the reason why the albinic phenotype was not observed in seedlings when grown in a paddy field. Therefore, *WP2* is essential for chloroplast development under high‐temperature conditions.

### Positional cloning of *WP2*

**Fig. 2:**
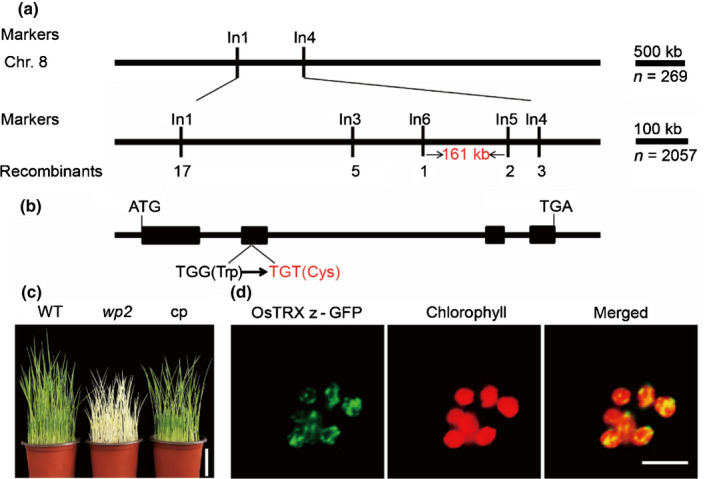
Map‐based cloning of *WP2*. (a) *WP2* was initially mapped to chromosome 8 and subsequently and fine‐mapped to a 161‐kb region between the InDel markers In6 and In5. (b) Diagram of *LOC_Os08g29110* gene structure showing that a single nucleotide substitution results in a substitution of a tryptophan residue by a cysteine residue in *wp2*. Lines indicate introns and black boxes represent exons. (c) Phenotypic comparison of 10‐d‐old seedlings of rice wild‐type, *wp2* mutant and complemented plants (cp) grown at 35°C. (d) Localisation of OsTRX z‐GFP‐fusion protein in rice protoplasts. Bars: (c) 6 cm; (d) 10 µm.

Initial location of the *WP2* locus in chromosome 8 was determined using 269 mutant F_2_ individuals from a cross between the *wp2* mutant and IR36 (*indica* variety) were used for initial mapping. Further delimitation was made to a 161‐kb region between markers In5 and In6 using 2057 F_2_ recessive individuals (Fig. 2a). Forty‐five open reading frames (ORFs) were predicted in the region (http://www.gramene.org/). Sequencing analysis showed that only the ORF of *LOC_Os08g29110* encoding a putative thioredoxin protein was different between the wild‐type and *wp2* mutant. A single base transition (G→T) caused substitution of a tryptophan residue (111) by a cysteine residue next to the conserved CXXC motif (Figs 2b, S2). To verify that this mutation was responsible for the *wp2* mutant phenotype, the coding sequence of *LOC_Os08g29110* driven by the CaMV35S promoter was transformed into the *wp2* mutant. All positive transgenic lines had a rescued green phenotype when grown at 35°C (Fig. 2c). In addition, three *LOC_Os08g29110* knockout lines from a previous study (He *et al.*, 2018) showed an albino seedling phenotype at both 25 and 35°C (Fig. S3). These results indicated that *LOC_Os08g29110* was the gene responsible for the *wp2* phenotype and that *wp2* was a mutation of *LOC_Os08g29110*.

Amino acid sequence analysis showed that WP2 was orthologous to AtTRX z (*At3g06730*) and was highly conserved in flowering plants (Fig. S2). *WP2* was renamed *OsTRX z* to conform with standard nomenclature. qRT‐PCR analysis revealed that *OsTRX z* was mainly expressed in green tissues including leaves, stems, leaf sheaths, panicles and seedling tissues, but expression was much lower in roots (Fig. S4a). Given the temperature‐dependent phenotype of *wp2*, transcript levels of *OsTRX z* were examined in plants grown at different temperatures. The transcript level of *OsTRX z* was induced by continuous high temperature or by high‐temperature shift treatments (Fig. S4b,c). The protein level of OsTRX z was also induced by high temperature (Fig. S4d). Subcellular localisation assay revealed that OsTRX z‐GFP‐fusion protein (with the green fluorescence protein fused to the C‐terminus of OsTRX z) exhibited a punctate localisation pattern in chloroplasts (Fig. 2d).

### OsTRX z is essential for transcription of plastid‐encoded genes

**Fig. 3:**
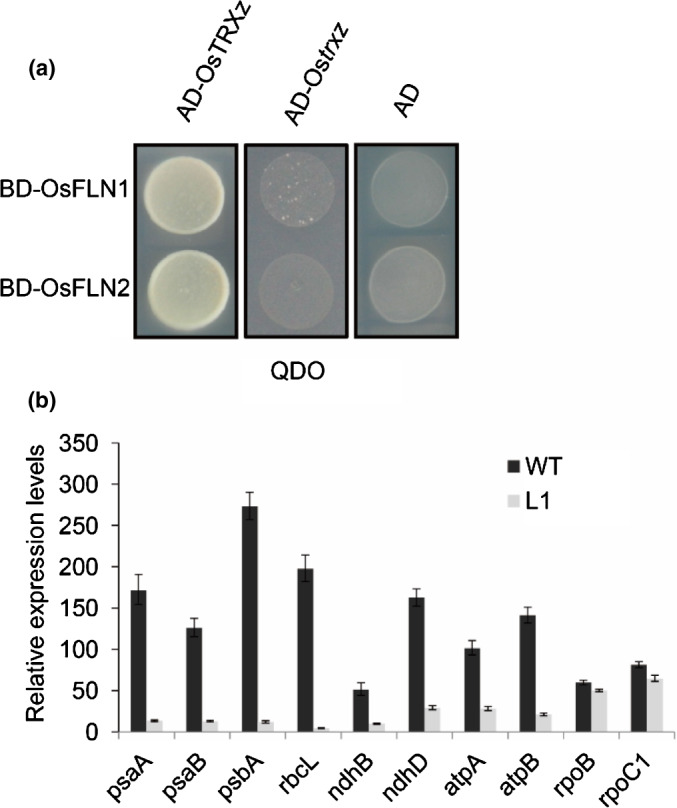
OsTRX z is essential for PEP activity. (a) Yeast‐two‐hybrid assay showing that OsTRX z but not Os*trx z*, interacts with OsFLN1 **(** *LOC_Os01g63220* **)** and OsFLN2 **(** *LOC_Os03g40550* **)**. (b) Relative expression levels of plastidial‐encoded genes in 10‐d‐old rice wild‐type and L1 seedlings grown at 35°C. Values are means ± SD of three biological replicates.

Chloroplast genes are transcribed by nuclear‐encoded polymerase (NEP) and PEP. NEP is responsible for the transcription of genes encoding plastidic PEP subunits, ribosomal proteins and other plastidic ‘housekeeping’ proteins. PEP transcribes genes involved in the formation of photosynthetic machinery (Shiina *et al.*, 2005). Previous studies in Arabidopsis showed that AtTRX z interacts with AtFLN1 and AtFLN2 and is a component of PEP (Arsova *et al.*, 2010). Yeast‐two‐hybrid assay (Y2H) showed that OsTRX z also interacted with OsFLN1 and OsFLN2 (Fig. 3a). Investigation of plastid‐encoded genes in wild‐type and *Ostrx z* knockout lines showed that transcription of photosynthesis‐related genes (*psaA*, *psaB*, *psbA* and *rbcL*) was significantly decreased in *Ostrx z* knockout lines but genes (*rpoB* and *rpoC1*) transcribed by NEP were barely changed (Fig. 3b). These results suggested that OsTRX z was essential for PEP activity.

### OsTRX z interacts with OsMORFs

**Fig. 4:**
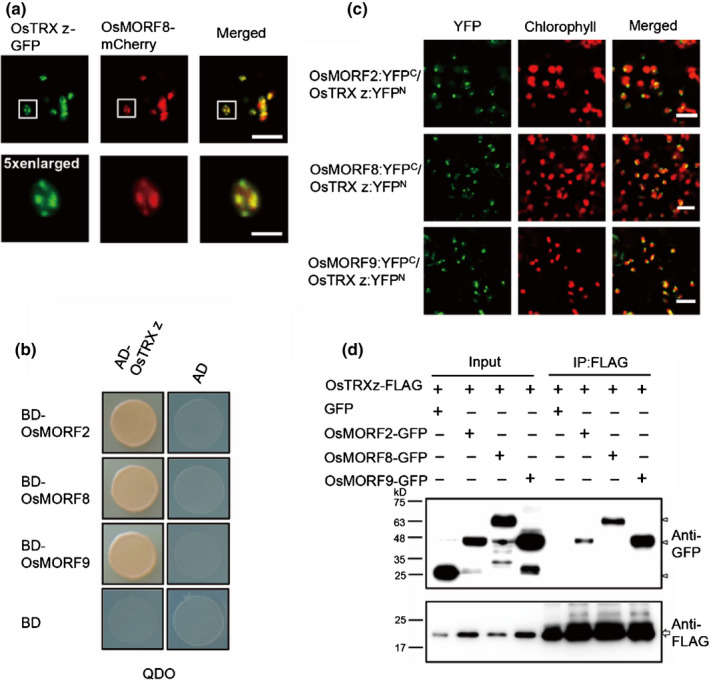
OsTRX z interacts with OsMORFs *in vivo* and *in vitro*. (a) Co‐localisation of OsTRX z‐GFP and OsMORF8‐mCherry in rice protoplasts. The lower panel shows enlarged images corresponding to the boxed area in the upper panel. (b) Y2H assays showing that OsTRX z interacts with OsMORF2, OsMORF8 and OsMORF9. QDO indicates SD/−Trp−Leu−His−Ade dropout plate. (c) BiFC assays showing that OsTRX z interacts with OsMORF2, OsMORF8 and OsMORF9 in chloroplasts. (d) Co‐immunoprecipitation assays showing that OsTRX z‐FLAG precipitated OsMORF2‐GFP, OsMORF8‐GFP and OsMORF9‐GFP in the presence of 1 mM dithio‐bis(succinimidyl propionate) (DSP). Arrows indicate FLAG‐fusion proteins; Arrowheads indicate GFP or GFP‐fusion proteins. Bars: (a) upper panel 10 µm, lower panel 2 µm; (c) 20 µm.

**Fig. 5:**
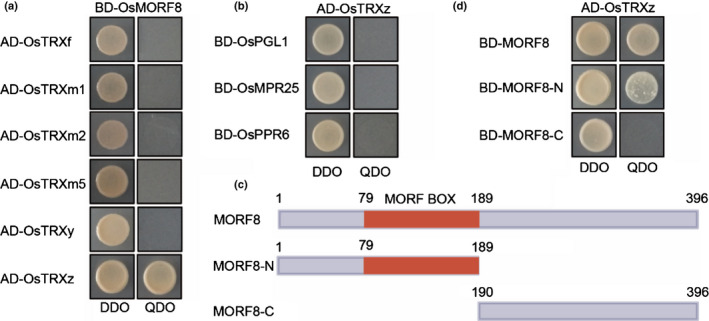
Y2H assays showing specific interactions between OsTRX z and MORF BOX. (a) Y2H assays showing that OsMORF8 interacts with OsTRX z, but not with other plastidial TRXs including OsTRX f (LOC_Os01g68480), OsTRX m1 (LOC_Os02g42700), OsTRX m2 (LOC_Os04g44830), OsTRX m5 (LOC_Os12g08730) and OsTRX y (LOC_Os01g73234). (b) Y2H assays showing that OsTRX z does not interact with OsPGL1 (LOC_Os12g06650), OsMPR25 (LOC_Os04g51530) and OsPPR6 (*LOC_Os05g49920*). (c) Diagram of the domain structure of OsMORF8 and its deletion forms. (d) Y2H assays showing that OsTRX z interacts with the N‐terminus of OsMORF8 which contains the MORF box.

Many proteins involved in different biological pathways are regulated by TRXs, but the numbers of TRX proteins are limited (Balmer *et al*., 2003, 2006; Wong *et al.*, 2004), therefore a single TRX might have multiple targets. To determine whether there were other targets of TRX z, we performed GO enrichment analyses of the first 100 co‐expression genes of *OsTRX z* and *OsFLN1*. Rice FREND analysis showed that *OsFLN1* mainly co‐expressed with genes in the proteolysis pathway, whereas *OsTRX z* co‐expressed with genes involved in proteolysis and RNA processing (Fig. S5a,b). Given that *OsTRX z* co‐expressed with RNA processing genes and had high similarity to the white panicle phenotype of the *wsp1* mutant that affected a MORF (Zhang *et al.*, 2017), we inferred that OsTRX z might be functionally associated with OsMORF2/WSP1. In Arabidopsis there are nine MORF proteins, among which MORF2, MORF8 and MORF9 are localised to the chloroplast (Takenaka *et al.*, 2012). We cloned their rice orthologues including *OsMORF2* (*LOC_Os04g51280*), *OsMORF8* (*LOC_Os09g33480*) and *OsMORF9* (*LOC_Os08g04450*). Like their *Arabidopsis* counterparts, these proteins were localised to the chloroplast with a punctate localisation pattern (Fig. S6) and OsTRX z‐GFP was co‐localised with OsMORF8‐mCherry (Fig. 4a). Y2H assays showed that OsTRX z interacted with OsMORF2, OsMORF8 and OsMORF9 (Fig. 4b). BiFC assays confirmed these interactions. There was strong YFP fluorescence with punctate localisation pattern in chloroplasts when combinations of OsTRX z‐YFP^N^ and OsMORF2‐YFP^C^, OsMORF8‐YFP^C^ or OsMORF9‐YFP^C^ were co‐expressed, indicating that OsTRX z interacts with chloroplast OsMORFs *in vivo* (Figs 4c,S7). *In vivo* co‐immunoprecipitation (Co‐IP) assay also confirmed these interactions (Fig. 4d). Most of the rice chloroplast TRXs and chloroplast PLS‐type PPRs, which function in site‐specific chloroplast RNA editing were selected to test for specific interaction between OsTRX z and OsMORFs (Toda *et al.*, 2012; Tang *et al.*, 2017; Xiao *et al.*, 2018). Y2H results showed no interaction between other chloroplast TRXs and OsMORF8 nor interaction of OsTRX z with PLS‐type PPRs (Fig. 5a,b). BiFC and Co‐IP assays detected no interaction between OsTRX z with the PLS‐type PPRs (Fig. S8a,b). Domain deletion analysis showed that the N‐terminal MORF box of OsMORF8 was responsible for interaction with OsTRX z (Fig. 5c,d).

TRX proteins usually interact with target proteins in a redox‐dependent manner (Meyer *et al.*, 2005). To test whether the interaction between OsTRX z and chloroplast OsMORFs was regulated by redox, *in vivo* co‐immunoprecipitation (Co‐IP) assays showed that the interaction between OsTRX z and OsMORF8 was very weak (Fig. S9a), but that the strength of interaction was significantly enhanced by addition of the crosslinking agent, dithio‐bis(succinimidyl propionate) (DSP) (Fig. 4d). Moreover, the interaction between OsTRX z and OsMORF8 was disrupted by the strong reducing agent dl‐dithiothreitol (DTT) both *in vivo* and *in vitro* (Fig. S9a,b). Taken together, these results suggested that OsTRX z interacted with OsMORFs in a redox‐dependent manner.

### Formation of OsMORF heterodimers is regulated by their redox state

**Fig. 6:**
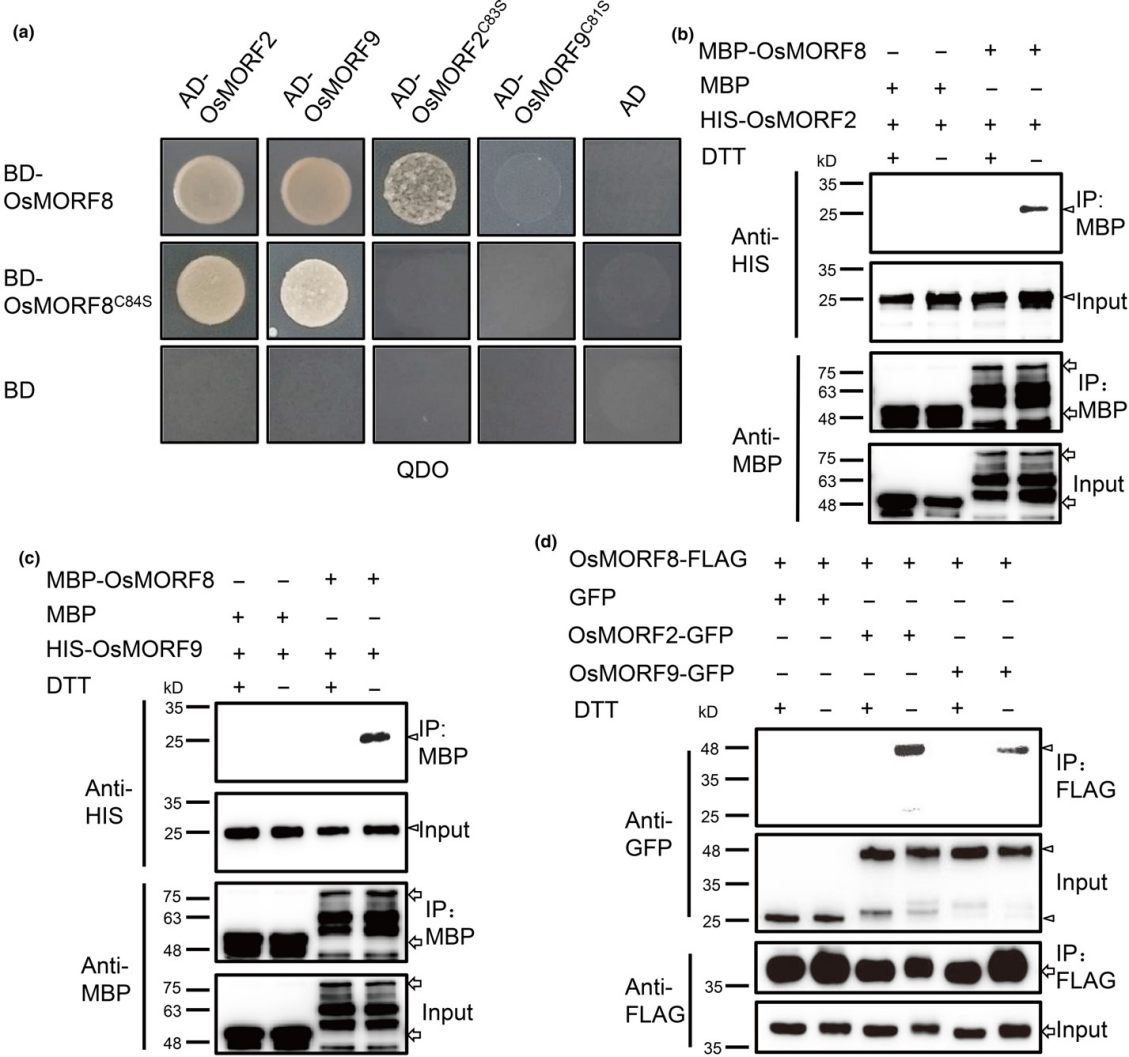
Redox regulation of the OsMORF–OsMORF interactions. (a) Y2H assays showing interactions between OsMORF8, OsMORF2 and OsMORF9. The Cys to Ser mutation disrupts their interactions. (b, c) *In vitro* pull‐down assays of recombinant HIS‐OsMORF2 (b) or HIS‐MORF9 recombinant proteins (c) using resins coupled to MBP‐OsMORF8 treated with or without DTT. Arrows indicate MBP and MBP‐fusion proteins; Arrowheads indicate HIS‐fusion protein. (d) Co‐immunoprecipitation assay showing that OsMORF2‐GFP and OSMORF9‐GFP are co‐precipitated by OsMORF8‐FLAG, and that treatment with DTT abolishes their interaction. Arrows indicate FLAG‐fusion proteins; Arrowheads indicate GFP‐fusion proteins.

MORF proteins in Arabidopsis interact with each other to form homodimers and heterodimers (Zehrmann *et al.*, 2015). Similarly, rice OsMORF2/WSP1 interacts with OsMORF8 (Zhang *et al.*, 2017). A recent study showed that the larger mass fractions (*c.* 413–670 kDa) of *Zea mays* ZmMORF9 exhibited higher RNA editing activity than the small mass fractions (*c.* 158 kDa), suggesting that interactions between MORFs are essential for RNA editing activity (Sandoval *et al.*, 2019). As the basic function of thioredoxins is to regulate the cysteine (Cys) redox state of target proteins (Arner & Holmgren, 2000), we wondered whether the interaction between OsMORF proteins is regulated by the redox state of the conserved Cys. Amino acid sequence alignment showed that OsMORF2, OsMORF8 and OsMORF9 shared a conserved Cys residue in their MORF box with all other MORFs in Arabidopsis and rice (Fig. S10). Y2H assays of OsMORFs and variants with mutations in the conserved Cys residue showed that mutation of the conserved Cys to Ser significantly weakened the interactions between OsMORFs (Fig. 6a). Treatment with the strong reducing agent DTT abolished the interactions of OsMORF8 with OsMORF2 and OsMORF9 in the *in vitro* pull‐down assays (Fig. 6b,c). Co‐IP assays confirmed results *in vivo* (Fig. 6d). An earlier study also showed that MORFs interacted with PLS‐PPRs and might be important for organelle RNA editing (Takenaka *et al.*, 2012). Further tests of whether the Cys redox state of MORFs affected their interactions with PLS‐type PPR proteins showed that the Cys→Ser mutation did not affect those interactions (Fig. S11). These results suggested that oxidised MORFs, but not reduced MORFs have the capacity to interact with each other to form heterodimers.

### *TRX z* is essential for chloroplast RNA editing

**Fig. 7:**
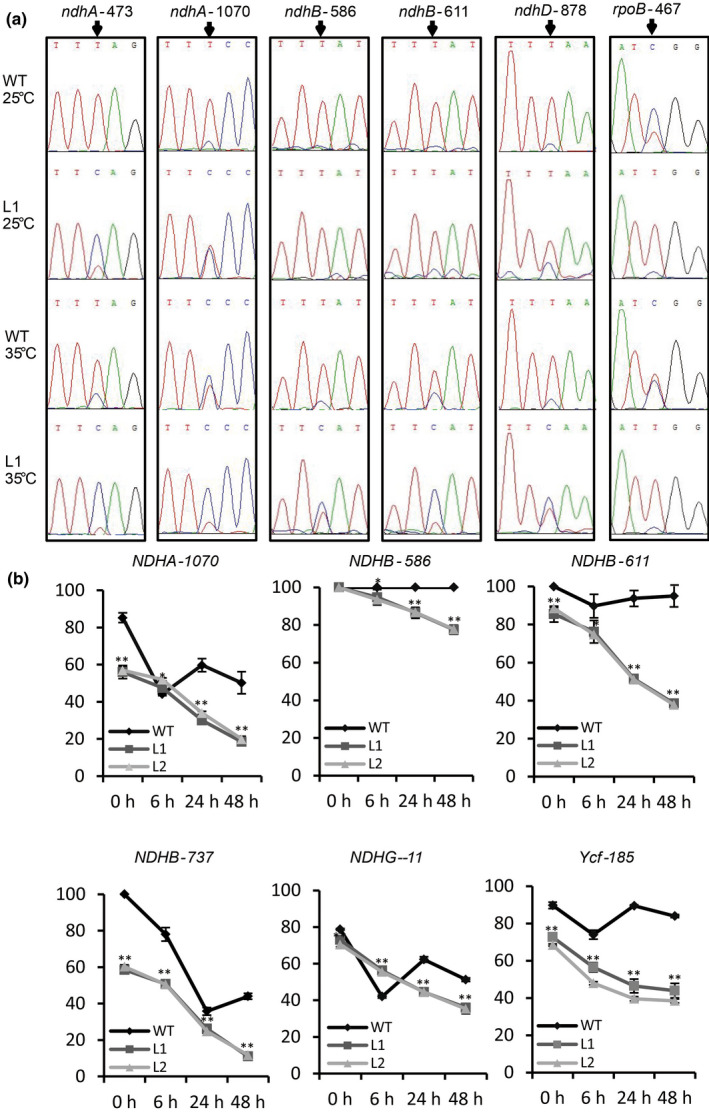
OsTRX z affects chloroplast RNA editing at multiple sites under high temperatures. (a) Sequencing analyses showing that RNA editing in chloroplasts is regulated by temperature and that the editing efficiencies of several plastidial‐encoded genes are affected in L1. (b) RNA editing efficiency analyses of *NDHA‐1070*, *NDHB‐586*, *NDHB‐611*, *NDHB‐737*, *NDHG‐‐11* and *Ycf3‐185* after transfer from 25 to 35°C in wild‐type and two CRISPR lines of *OsTRX z*. The rice plants were grown at 25°C for 10 d and then transferred to 35°C. Error bars indicate SD based on four biological replicates. *, *P* < 0.05; **, *P* < 0.01.

Considering the interactions between OsTRX z and OsMORFs, we tested whether the chloroplast RNA editing efficiencies were altered in the *Ostrx z* mutant. Sequencing analysis was used to detect the RNA editing efficiencies at all known chloroplast RNA editing sites, the results showed that *Ostrx z* knockout lines exhibited significant difference in chloroplast RNA editing compared with the wild‐type at 25°C (Fig. 7a; Table S1). In consideration of the high‐temperature albino of *wp2* and high‐temperature‐induced expression of *OsTRX z*, a test of the RNA editing efficiencies of wild‐type and *Ostrx z* knockout lines at 35°C showed that *Ostrx z* knockout lines had more significant chloroplast RNA editing changes. The editing efficiencies of 14 of the 23 known chloroplast RNA editing sites were reduced, whereas the editing efficiencies of *rpoB*, *rps8* and *ndhF* were increased in Ostrx z knockout lines at 35°C (Fig. 7a; Table S1). Tests of the plastid RNA editing efficiencies in the *wp2* mutant also showed significant changes at 35°C, but no differences from the wild‐type at 25°C (Fig. S12; Table S1). RNA editing defects were rescued in *35S:OsTRX z* transgenic plants (Fig. S12; Table S1). These results suggested that OsTRX z is involved in chloroplast RNA editing and is especially required at high temperature. To further test this notion, we conducted a temperature shift experiment using wild‐type and *Ostrx z* knockout lines grown at 25°C for 10 d and then transferred to 35°C. RNA editing efficiencies initially decreased and then increased slightly in the wild‐type plants, but continued to decrease in the *Ostrx z* knockout lines (Fig. 7b). These results indicated that OsTRX z was essential for chloroplast RNA editing under high temperature.

**Fig. 8:**
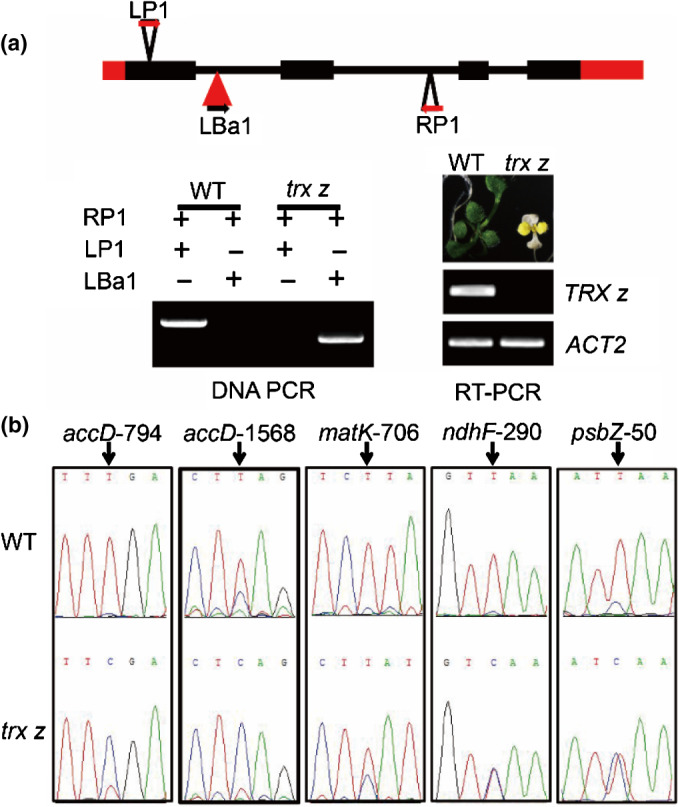
RNA editing analysis of the Arabidopsis *trx z* mutant. (a) Phenotype of 7‐d‐old Arabidopsis *trx z* mutant (Salk_028162C) and PCR verification of the T‐DNA insertion mutation. The T‐DNA insertion site (indicated by a red triangle) and locations of the primers (LP1, RP1 and LBa1) are indicated. The black and red boxes indicate exons and untranslated regions (UTRs), respectively. The black lines represent introns. *TRX z* transcripts were detected by RT‐PCR with gene‐specific primers. *ACTIN* (*ACT2*) was used as an internal control. (b) RNA editing efficiency analyses of several affected chloroplast editing sites in wild‐type and *trx z* mutant.

To further confirm that TRX z was involved in chloroplast RNA editing, we obtained an Arabidopsis *trx z* mutant from the SALK collection (Salk_028162C). We first confirmed that Salk_028162C was a knockout mutant of AtTRX z (Fig. 8a). Sequencing showed that the Arabidopsis *trx z* mutant also exhibited significant changes in chloroplast RNA editing (Fig. 8b; Table S2) suggesting that *TRX z* was essential for plastid RNA editing in plants.

Previous studies have shown that some albinic Arabidopsis mutants exhibited mild RNA editing defects (Kakizaki *et al.*, 2012; Tseng *et al.*, 2013). To clarify the relationship between the albinic phenotype and changed RNA editing, we examined the plastid RNA editing efficiencies of a previously reported *wsl3* mutant (mutant of *OspTAC3*) (Wang *et al.*, 2016). Although the *wsl3* mutant had an albinic phenotype at 25°C (Fig. S13a), the RNA editing efficiencies were essentially normal relative to the wild‐type (93‐11) except for a small increase in RNA editing of *rpoB* (Fig. S13b; Table S3). This suggested that the albinic phenotype of the *wsl3* mutant was not caused by defective RNA editing. These observations suggested that the broad‐spectrum plastid RNA editing defect observed in the *Ostrx z* mutants was unlikely to have been caused by loss of PEP activity.

## Discussion

### TRX z directly participates in chloroplast RNA editing

Genetic analysis of the high‐temperature‐sensitive albinic *wp2* mutant showed that the mutant phenotype was caused by a single base transition in a chloroplast thioredoxin z gene (Figs 1, 2). Knockout mutants of OsTRX z were albinic at both 25°C and 35°C (Fig. S3). We presumed that the *wp2* mutant had a weaken form of OsTRX z. We found that the rice *Ostrx z* mutants were changed in chloroplast RNA editing at multiple sites especially at high temperature, suggesting that *OsTRX z* was essential for chloroplast RNA editing in a temperature‐dependent manner (Figs 7, S12; Table S1). TRXs are important regulatory proteins in all organisms and TRX systems are especially complex in plants. According to previous studies, a single TRX might have multiple targets (Balmer *et al*., 2003, 2006; Wong *et al.*, 2004). Previous studies have reported that TRX z is a component of plastid transcriptionally active chromosomes (pTACs) and that it interacts with AtFLN1 and AtFLN2 (Arsova *et al.*, 2010), as well as various other chloroplast proteins, including CHLI, TSV and PRIN2 (Zhang *et al.*, 2015; Sun *et al.*, 2017; Diaz *et al.*, 2018), suggesting that TRX z may also play a multifaceted role in regulating chloroplast development and function. Consistent with previous studies, we showed that OsTRX z interacts with OsFLN1 and OsFLN2 and regulates the expression of plastid‐encoded genes during chloroplast development (Fig. 3). Mutations of thioredoxins usually lead to reduced target activity rather than loss of target activity (Wang *et al.*, 2013; Daloso *et al.*, 2015). We speculated that partial RNA editing impairment of *Ostrx z* might not lead to an albino phenotype, and that the albinic phenotype of *Ostrxz* and *wp2* mutants could be caused by a series of reduced chloroplast target activities of OsTRX z.

Previous studies have shown that some albinic mutants in Arabidopsis had chloroplast RNA editing defects (Kakizaki *et al.*, 2012; Tseng *et al.*, 2013), raising the question whether defective RNA editing might cause the albinic phenotype. Some studies, however, have suggested that there was no correlation between RNA editing defects and albinism. RNA editing defects and albinism in Arabidopsis *ppo1* mutant were unrelated (Zhang *et al.*, 2014). Similarly, the albino *wsl3* mutant in rice showed almost no impairment in RNA editing (Fig. S13; Table S3). In further support of the proposition, an earlier study of an albinic mutant showed that a change in the *OsPPR6* gene, which encodes a PLS‐type PPR protein, caused a single site‐specific RNA editing defect in *NDHB‐737*. The editing defect was thought not to be responsible for the albinic phenotype (Tang *et al.*, 2017). Other studies on chlorotic mutants *wsl4* and *wsl5* in rice also showed minimal effects on chloroplast RNA editing (Wang *et al.*, 2017; Liu *et al.*, 2018). We used three lines of evidence to support the notion that TRX z is directly involved in RNA editing. First, we showed that chloroplast RNA editing efficiencies were largely normal in the *wsl3* mutant but significantly changed in *Ostrx z* knockout plants (Tables S1, S3), although both TRX z and WSL3 are components of the pTAC complex. Second, we showed that the *Attrx z* mutant exhibited severe RNA editing defects as reported for chloroplast *Atmorf* mutants (Takenaka *et al.*, 2012), whereas the RNA editing efficiencies of some sites (including *matK‐640*, *psbE‐214*, *psbF‐77* and *psbZ‐50*) were not affected in other albinic mutants (Kakizaki *et al.*, 2012; Tseng *et al.*, 2013), but affected in Arabidopsis *trx z* and *morf* mutants (Fig. 8). Thirdly, we demonstrated that TRX z directly interacted with all chloroplast OsMORFs through their MORF‐boxes (Figs 4, 5). Based on these results, we concluded that the editing defects in *Ostrxz* mutant were caused by mutation in *OsTRX z* itself rather than indirectly caused by the albino phenotype. Our results suggested that TRX z directly participates in chloroplast RNA editing, which is one of the functions of TRX z. Moreover, TRX z is conserved in flowering plants (with chloroplast RNA editing) and does not exist in algae (without chloroplast RNA editing) (Cahoon *et al.*, 2017), providing support for the proposal that TRX z is involved in chloroplast RNA editing.

### OsTRX z affects the formation of heterodimers of OsMORFs by regulating the redox state of the conserved Cys residue in the MORF box

The basic function of TRXs is to reduce the Cys residue of their target proteins through catalysing thiol disulfide interchanges. A previous study showed that AtTRX z possesses a disulfide reductase activity (Arsova *et al.*, 2010). Previous studies also showed that MORFs interact with each other to form homodimers or heterodimers that could be essential for assembly of activated editosomes (Takenaka *et al.*, 2012; Zehrmann *et al.*, 2015; Sandoval *et al.*, 2019). Here, we found that chloroplast OsMORF proteins were the targets of OsTRX z and that the redox state of OsMORFs is essential for their interactions (Figs 4, 5, 6). We propose a working model in which TRX z regulates the dynamic assembly of the chloroplast editosome (Fig. S14). When one editosome completes its RNA editing job at one site, it is dissolved through reduction of MORFs. RNA editing at another site would require the reassembly of a new editosome by recruiting re‐oxidised MORFs (probably by reactive oxygen species (ROS)) and another site‐specific PPR protein. This model explains the general effects of OsTRX z and MORFs on plastidial RNA editing and suggests a redox‐regulated dynamic assembly/disassembly process of the editosome. Consistent with our model, a recent study showed that overexpression of *AtMORF*2 led to chloroplast RNA editing defects (Zhao *et al.*, 2019), suggesting that homeostasis of MORFs is essential for their normal functions.

### Possible physiological significance of redox regulation in RNA editing for adaptation to stress conditions

RNA editing in plants has long been thought to be a mechanism for correcting defective organelle transcripts (Stern *et al.*, 2010; Sun *et al.*, 2016), however a satisfactory explanation for why and how such a system evolved has not been forthcoming. In polar octopuses, A→I RNA editing resulting in an amino acid substitution of K^+^ channel is essential for its temperature adaptation (Garrett & Rosenthal, 2012), suggesting that RNA editing plays an important role in environmental adaptation. In plants, high temperature, salt stress and fungal infection have been shown to influence chloroplast RNA editing (Nakajima & Mulligan, 2001; Karcher & Bock, 2002; Garcia‐Andrade *et al.*, 2013; Rodrigues *et al.*, 2017) and it is notable that RNA editing defects in *NDH* genes are beneficial to plant immunity (Garcia‐Andrade *et al.*, 2013). Mitochondrial RNA editing is affected by high temperature (Zhang *et al.*, 2019). Previous studies also reported that mitochondria PPR proteins affect mitochondria RNA editing and that mutation of these PPRs leads to increased tolerance to drought and salt stresses (Yuan & Liu, 2012; Zhu *et al.*, 2014). Together, these observations suggest that changes in RNA editing in plants might be an important adaptive response to biotic or abiotic stresses. It is known that, in plants, abiotic and biotic stresses usually lead to increase in ROS (Mittler *et al.*, 2004; Suzuki *et al.*, 2012; You & Chan, 2015; Choudhury *et al.*, 2017; Farooq *et al.*, 2019), which might in turn affect the redox status of TRXs and MORFs and the assembly of the editosome, eventually leading to changes in RNA editing efficiencies for an adaptive response. In support of this notion, a previous study in rice showed that mitochondria RNA editing was affected by oxidative stress (Xiong *et al.*, 2017). Most RNA editing sites are involved in electron transport complexes (mitochondria respiratory electron transport and chloroplast cyclic electron flow) that not only generate energy but also produce ROS (Dutilleul *et al.*, 2003; Wang *et al.*, 2006; Jacoby *et al.*, 2012). We conjectured that reduced RNA editing may help to reduce ROS production through decreased electron transport and thus improve plant fitness under stress conditions. Further experimental work is required to more robustly test this proposition. In previous studies, TRX z was found to participate in the redox regulation of transcription of chloroplast encoded genes (Arsova *et al.*, 2010; Diaz *et al.*, 2018). The dual function of TRX z in transcription and RNA editing might make plants more efficiently adaptable to variable chloroplast redox state through regulating chloroplast RNA.

## Author contributions

J‐MW, H‐YW and Y‐HW conceived and designed the project. Y‐LW, Y‐HW, Y‐LR, E‐CD, X‐PZ, Y‐YH, J‐PZ, R‐BC, JL, XT, Y‐YZ, DW, LJ, XZ, X‐PG, S‐JL, Y‐LT, XL and L‐MC performed the experiments; J‐MW, H‐YW, Y‐LW, Y‐HW and Y‐LR wrote the manuscript. Y‐LW, Y‐HW and Y‐LR contributed equally to this work.

## Supporting information


**Fig. S1** Phenotypes of the *wp2* mutant.
**Fig. S2** Amino acid sequence alignment of OsTRX z, Os*trx z* and their homologues.
**Fig. S3** Phenotype of the knockout mutant of *OsTRX z*.
**Fig. S4** Expression of OsTRX z at transcript and protein levels.
**Fig. S5** Function predicting of OsTRX z
**Fig. S6** Subcellular localisation of OsMORF2, OsMORF8 and OsMORF9 proteins.
**Fig. S7** Negative control of the BiFC assay.
**Fig. S8** OsTRX z does not interact with rice chloroplast PPRs *in vivo*.
**Fig. S9** DTT abolishes the interaction between OsTRX z and OsMORF8.
**Fig. S10** Partial amino acid sequence alignment of all MORFs in rice and Arabidopsis.
**Fig. S11** Y2H assays showing the interactions between OsMORF8, OsMORF8^C84S^ and PLS‐type PPRs.
**Fig. S12** Sequencing analyses showing the chloroplast RNA editing efficiencies of wild‐type, *wp2*, complemented plants (cp) at 25°C and 35°C.
**Fig. S13** Phenotype and chloroplast RNA editing levels of *wsl3*.
**Fig. S14** Model of TRX z regulation of plastidial RNA editing in plants.
**Table S1** Analysis of all known chloroplast editing sites in wild‐type, *wp2*, L1 and cp at 25°C and 35°C.
**Table S2** Analysis of all known *Arabidopsis* chloroplast editing sites in wild‐type and *trx z*.
**Table S3** Analysis of all known chloroplast editing sites in 93‐11 and *wsl3* at 25°C.
**Table S4** Primers used in this study.
Please note: Wiley Blackwell are not responsible for the content or functionality of any Supporting Information supplied by the authors. Any queries (other than missing material) should be directed to the *New Phytologist* Central Office.

## Data Availability

Sequence data for this article can be found in the GenBank library under the accession number: *OsTRX z* (*LOC_Os08g29110*), *AtTRX z* (*At3g06730*), *OsFLN1* (*LOC_Os01g63220*), *OsFLN2* (*LOC_Os03g40550*), *OsMORF2* (*LOC_Os04g51280*), *OsMORF8* (*LOC_Os09g33480*), *OsMORF9* (*LOC_Os08g04450*), *OsPPR6* (*LOC_Os05g49920*), *OsPGL1* (*LOC_Os12g06650*), *OsMPR25* (*LOC_Os04g51350*).
