# Supplementary material for: white panicle2 encoding thioredoxin z, regulates plastid RNA editing by interacting with multiple organellar RNA editing factors in rice
Source: New Phytol. 2020 Dec 19;229(5):2693–706. doi: 10.1111/nph.17047 (PMC8027827; doi:10.1111/nph.17047)
Supplement: Supplementary file 1 — Fig. S1 Phenotypes of the wp2 mutant. Fig. S2 Amino acid sequence alignment of OsTRX z, Ostrx z and their homologues. Fig. S3 Phenotype of the knockout mutant of OsTRX z. Fig. S4 Expression of OsTRX z at transcript and protein levels. Fig. S5 Function predicting of OsTRX z Fig. S6 Subcellular localisation of OsMORF2, OsMORF8 and OsMORF9 proteins. Fig. S7 Negative control of the BiFC assay. Fig. S8 OsTRX z does not interact with rice chloroplast PPRs in vivo. Fig. S9 DTT abolishes the interaction between OsTRX z and OsMORF8. Fig. S10 Partial amino acid sequence alignment of all MORFs in rice and Arabidopsis. Fig. S11 Y2H assays showing the interactions between OsMORF8, OsMORF8C84S and PLS‐type PPRs. Fig. S12 Sequencing analyses showing the chloroplast RNA editing efficiencies of wild‐type, wp2, complemented plants (cp) at 25°C and 35°C. Fig. S13 Phenotype and chloroplast RNA editing levels of wsl3. Fig. S14 Model of TRX z regulation of plastidial RNA editing in plants. Table S1 Analysis of all known chloroplast editing sites in wild‐type, wp2, L1 and cp at 25°C and 35°C. Table S2 Analysis of all known Arabidopsis chloroplast editing sites in wild‐type and trx z. Table S3 Analysis of all known chloroplast editing sites in 93‐11 and wsl3 at 25°C. Table S4 Primers used in this study. Please note: Wiley Blackwell are not responsible for the content or functionality of any Supporting Information supplied by the authors. Any queries (other than missing material) should be directed to the New Phytologist Central Office. [file NPH-229-2693-s001.pdf]

New Phytologist Supporting Information

Article title:

***White Panicle2* encoding thioredoxin z, regulates plastid RNA editing by interacting with Multiple Organellar RNA Editing Factors in rice**

Authors:

Yunlong Wang, Yihua Wang, Yulong Ren, Erchao Duan, Xiaopin Zhu, Yuanyuan Hao, Jianping Zhu, Rongbo Chen, Jie Lei, Xuan Teng, Yuanyan Zhang, Di Wang, Xin Zhang, Xiuping Guo, Ling Jiang, Shijia Liu, Yunlu Tian, Xi Liu, Liangming Chen, Haiyang Wang, Jianmin Wan

Article acceptance date: 16 October 2020.

The following Supporting Information is available for this article:

Fig. S1 Phenotypes of the *wp2* mutant.

Fig. S2 Amino acid sequence alignment of OsTRX z, Os*trx z* and their homologs.

Fig. S3 Phenotype of the knockout mutant of *OsTRX z*.

Fig. S4 Expression of OsTRX z at transcript and protein levels.

Fig. S5 Function predicting of OsTRX z

Fig. S6 Subcellular localization of OsMORF2, OsMORF8 and OsMORF9 proteins.

Fig. S7 Negative control of the BiFC assay.

Fig. S8 OsTRX z does not interact with rice chloroplast PPRs *in vivo*

Fig. S9 DTT abolishes the interaction between OsTRX z and OsMORF8.

Fig. S10 Partial amino acid sequence alignment of all MORFs in rice and *Arabidopsis*.

Fig. S11 Y2H assays showing the interactions between OsMORF8, OsMORF8^{C84S} and PLS-type PPRs.

Fig. S12 Sequencing analyses showing the chloroplast RNA editing efficiencies of

wild type, *wp2*, complemented plants (cp) at 25°C and 35°C.

Fig. S13 Phenotype and chloroplast RNA editing levels of *wsl3*.

Fig. S14 Model of TRX z regulation of plastidial RNA editing in plants.

Table S1 Analysis of all known chloroplast editing sites in wild type, *wp2*, L1 and cp at 25°C and 35°C.

Table S2 Analysis of all known *Arabidopsis* chloroplast editing sites in wild type and *trx z*.

Table S3 Analysis of all known chloroplast editing sites in 93-11 and *wsl3* at 25°C.

Table S4 Primers used in this study

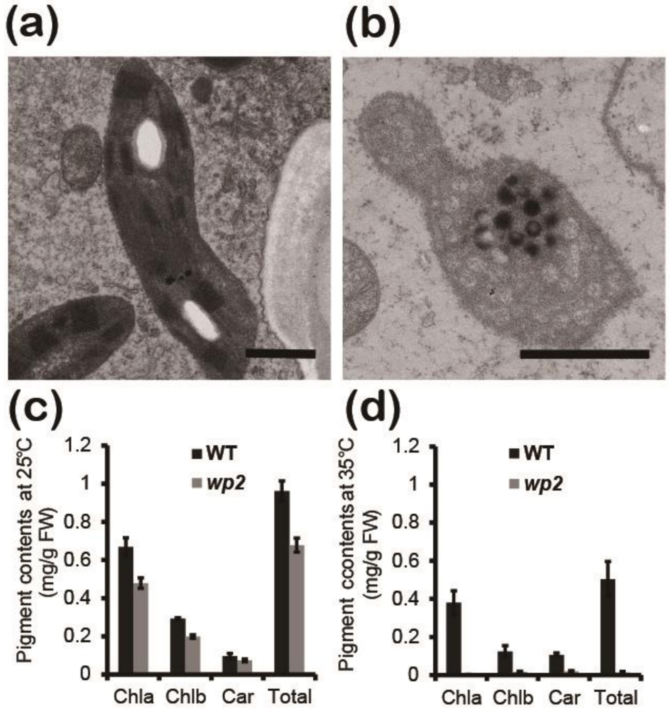

Fig. S1 Phenotypes of the *wp2* mutant. (a, b) Ultrastructure of chloroplasts in wild-type (a) and *wp2* mutant (b) panicles. (c, d) Pigment contents of 10-d old wild-type and *wp2* mutant seedlings grown at 25°C (c) and 35°C (d) respectively. Error bars indicate ±SD (n= 5) (c, d) FW = fresh weight. Bars: (a, b) 1μm.

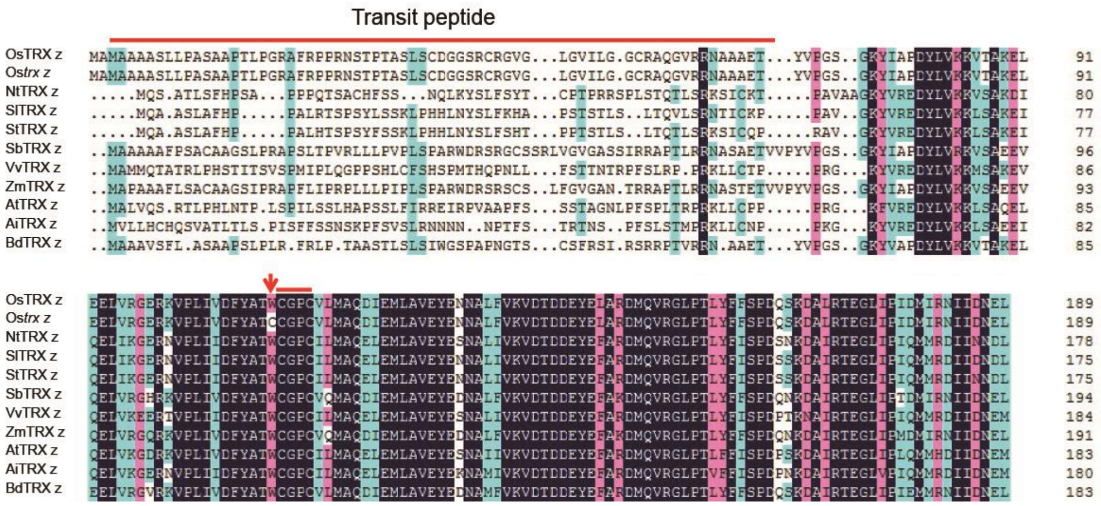

Fig. S2 Amino acid sequence alignment of OsTRX z, Os*trx z* and their homologs. The long red line shows the chloroplast transit peptide. The short red line shows the conserved CXXC motif (where X is any amino acid) in TRXs. The red arrow indicates the mutation site in *wp2*. The following sequences were used to establish the alignment: *Oryza sativa* (Os), OsTRX z, XP_015648902.1; *wp2*, Ostrx z; *Nicotiana tabacum* (Nt), NtTRX z, XP_016485759.1; *Solanum lycopersicum* (Si), SiTRX z NP_001234469.1; *Solanum tuberosum* (St), StTRX z, XP_006347665.1; *Sorghum bicolor* (Sb), SbTRX z, XP_002445439.1; *Vitis vinifera* (Vv), VvTRX z, XP_002264063.1; *Zea mays* (Zm), ZmTRX z, NP_001148388.1; *Arabidopsis thaliana* (At), AtTRX z, NP_187329.1; *Arachis ipaensis* (Ai), AiTRX z, XP_016182294.1; *Brachypodium distachyon* (Bd), BdTRX z, XP_003574431.1.

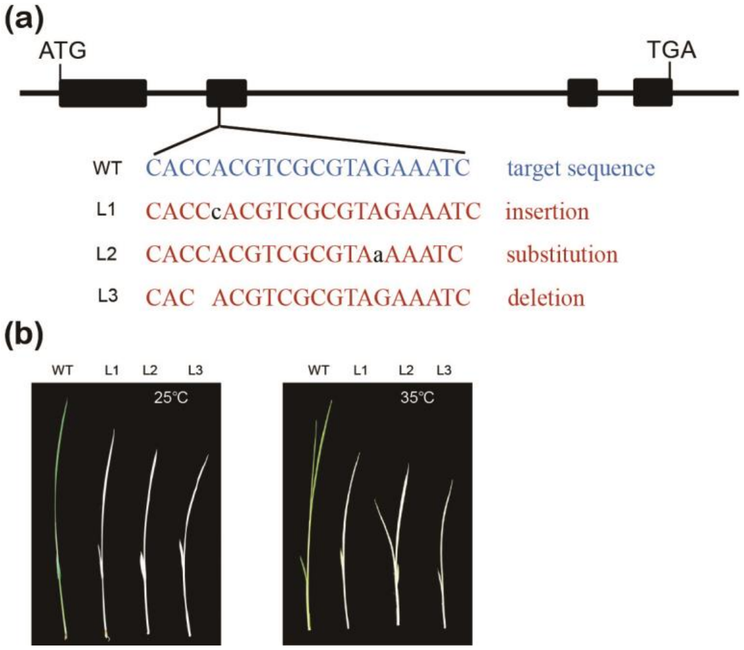

Fig. S3 Phenotype of the knockout mutant of *OsTRX z*. (a) Confirmation of the *OsTRX z* sequence variations in three lines (L1 to L3) generated using CRISPR/Cas9 technology. The PAM (Proto-spacer adjacent motif) sequence is in blue and variations in target sequence are in red. (b) Phenotype of 10-d old seedlings of wild-type and three knockout mutant lines of *OsTRX z* grown at 25°C and 35°C.

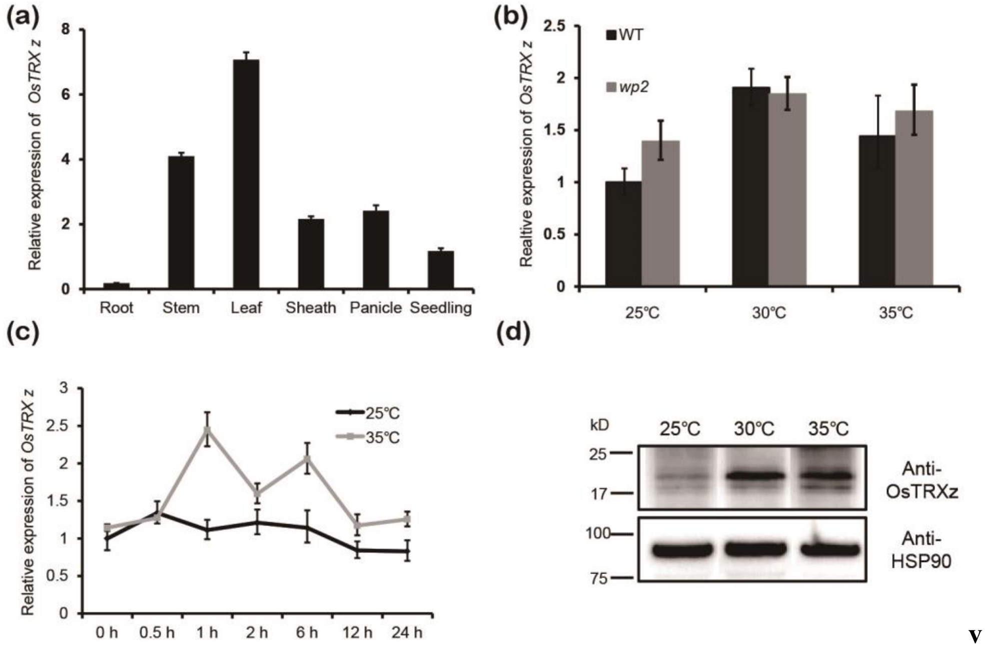

v

Fig. S4 OsTRX z accumulation at transcript and protein levels. (a) Relative expression of *OsTRX z* in different tissues, including root, stem, leaf, leaf sheath, panicle and seedling. (b) Relative expression of *OsTRX z* in 10-d old wild-type and *wp2* mutant seedlings grown at 25°C, 30°C and 35°C, respectively. (c) Relative expression of *OsTRX z* in wild-type seedlings grown at 25°C for 10 d and then grown at 35°C for 24 h with continuous illumination. Plants were treated with continuous illumination for two days before transfer to 35°C. (d) Protein levels of OsTRX z in wild-type seedlings grown at 25°C, 30°C and 35°C for 10 d. Values are means ± SD of three replicates. *UBIQUITIN* was used as an internal control for quantitative RT-PCR analysis. HSP90 was used as a loading control.

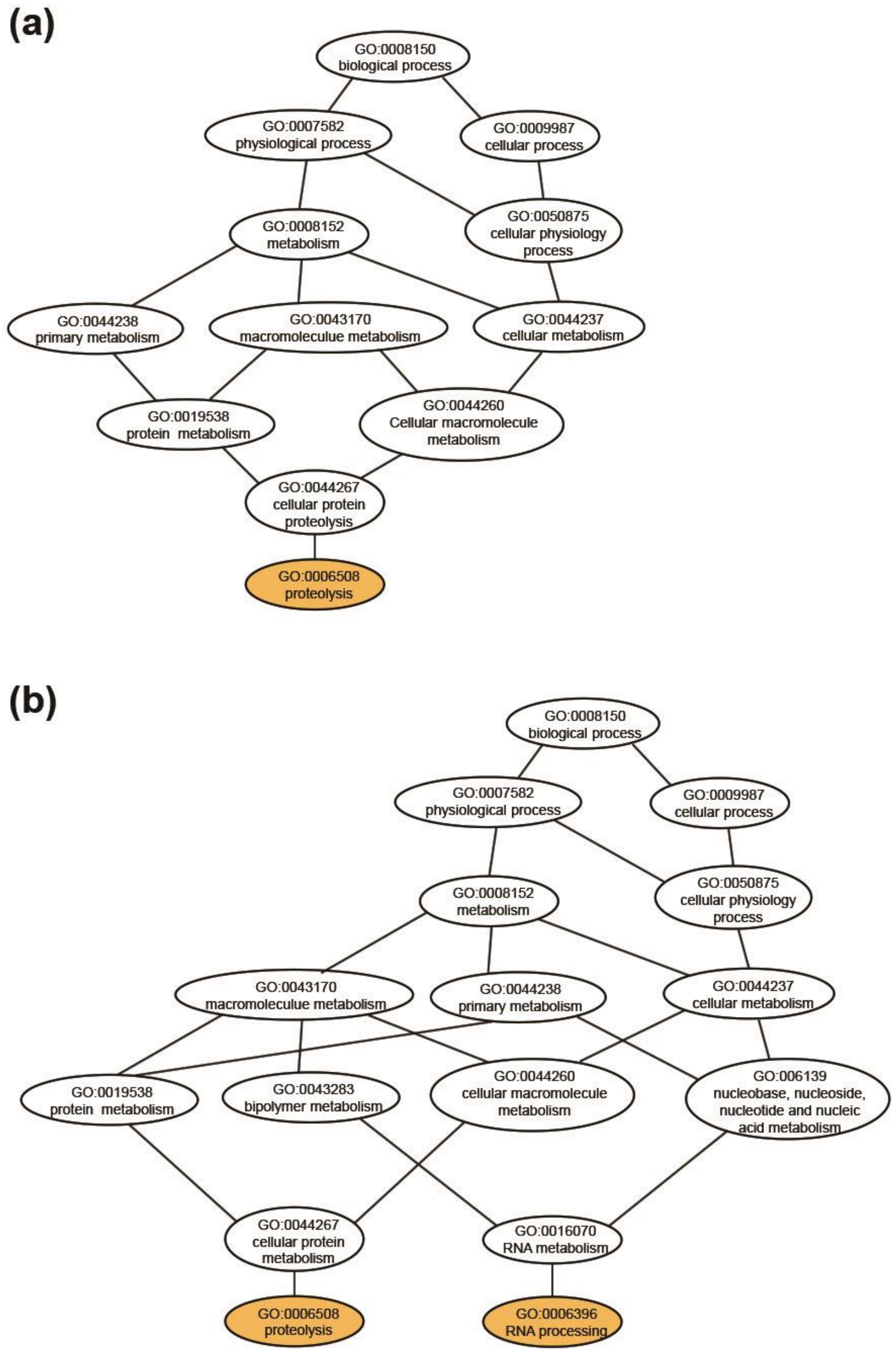

Fig. S5 Prediction of OsTRX z function. (a, b) GO enrichment analysis of first 100 co-expression of OsFLN1 (a) and OsTRX z (b). These analyses were performed with the Rice FREND (http://ricefrend.dna.affrc.go.jp/single-guide-gene.html).

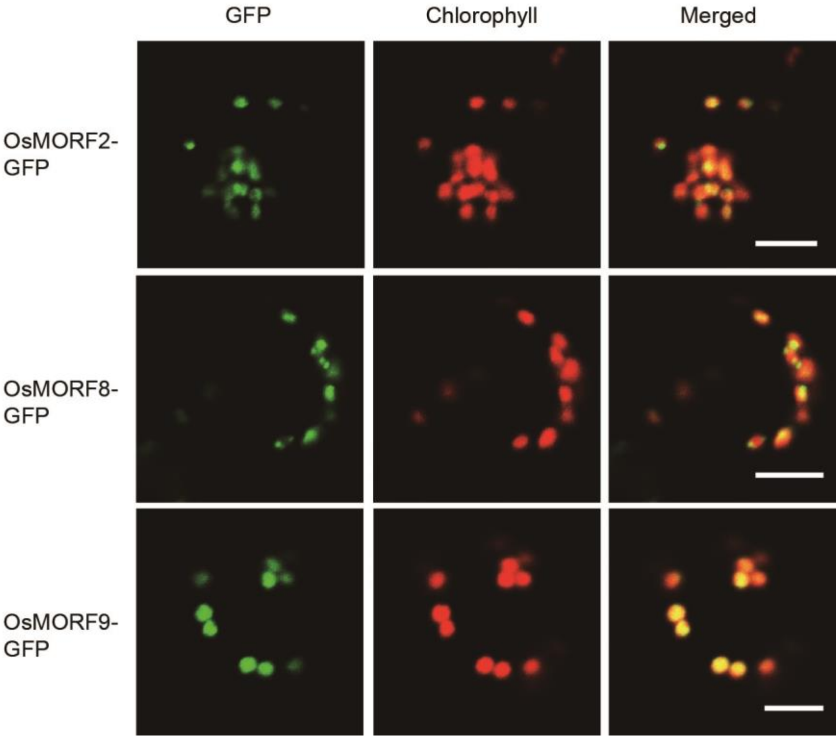

Fig. S6 Subcellular localization of the OsMORF2, OsMORF8 and OsMORF9 proteins. OsMORF2-GFP, OsMORF8-GFP and OsMORF9-GFP fusion proteins were localized to the chloroplasts in rice protoplasts. Bars: 10 μm.

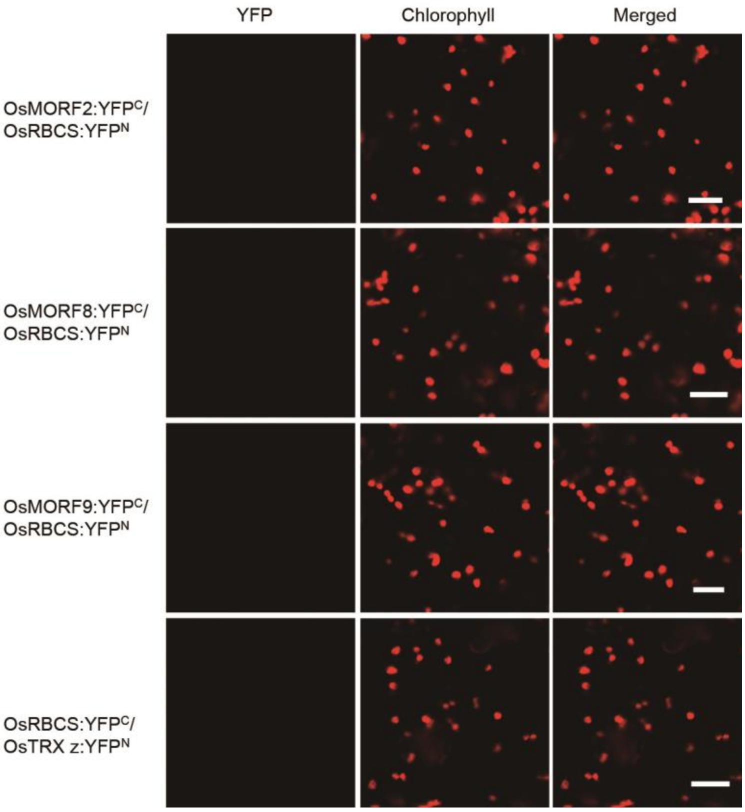

Fig. S7 Negative control of the BiFC assay. The chloroplast Ribulose-1,5-bisphosphate oxygenase small subunit (RbcS) was used as a negative control in the BiFC assay. No fluorescence signal was observed when OsRbcS-YFP^{N}/YFP^{C} was co-expressed with OsMORF2-YFP^{C}, OsMORF8-YFP^{C}, OsMORF9-YFP^{C} or OsTRX z-YFP^{N}. Bars: 20 µm.

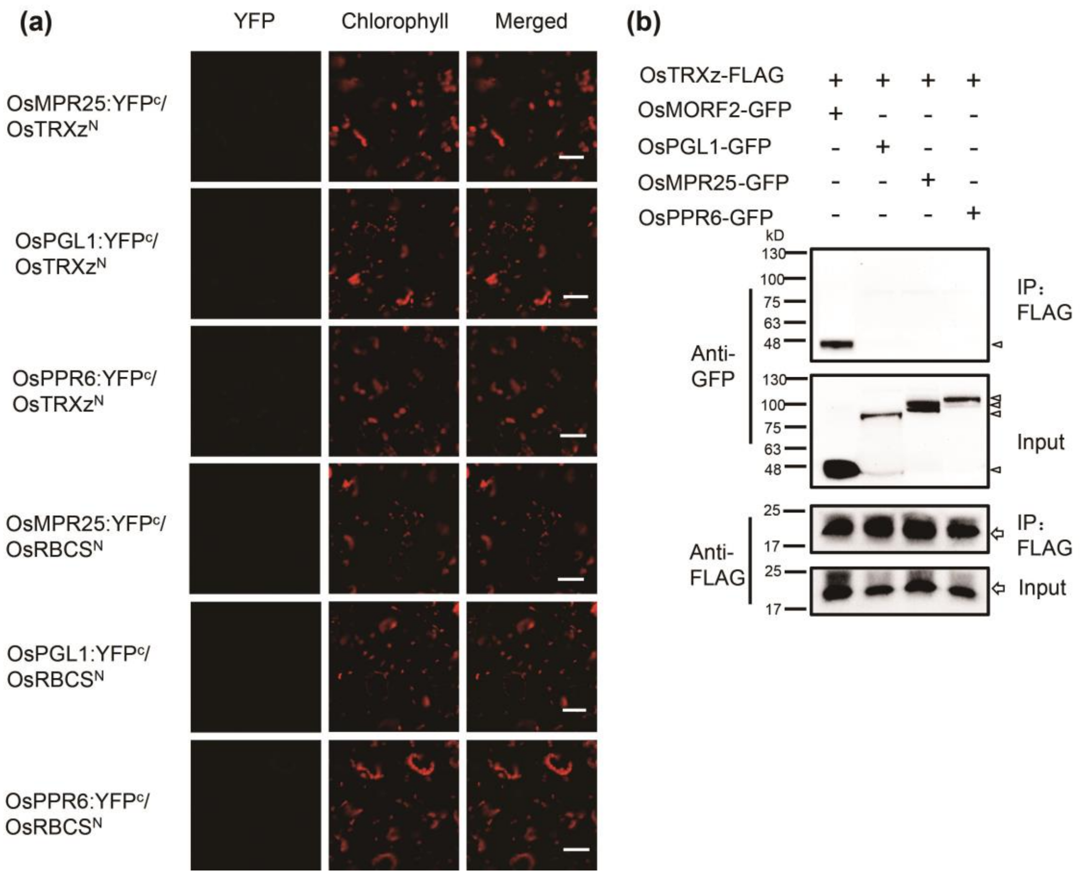

Fig. S8 OsTRX z does not interact with rice chloroplast PPRs *in vivo*. (a) BiFC assays showing that OsTRX z does not interact with OsPGL1, OsMPR25 and OsPPR6 in chloroplasts. (b) Co-immunoprecipitation assays showing that OsTRX z-FLAG precipitated OsMORF2-GFP but not OsPGL1-GFP, OsMPR25 and OsPPR6-GFP in the presence of 1 mM dithio-bis(succinimidyl propionate) (DSP). Arrows indicate FLAG-fusion proteins; Arrowheads indicate GFP-fusion proteins. Bars: 20 µm.

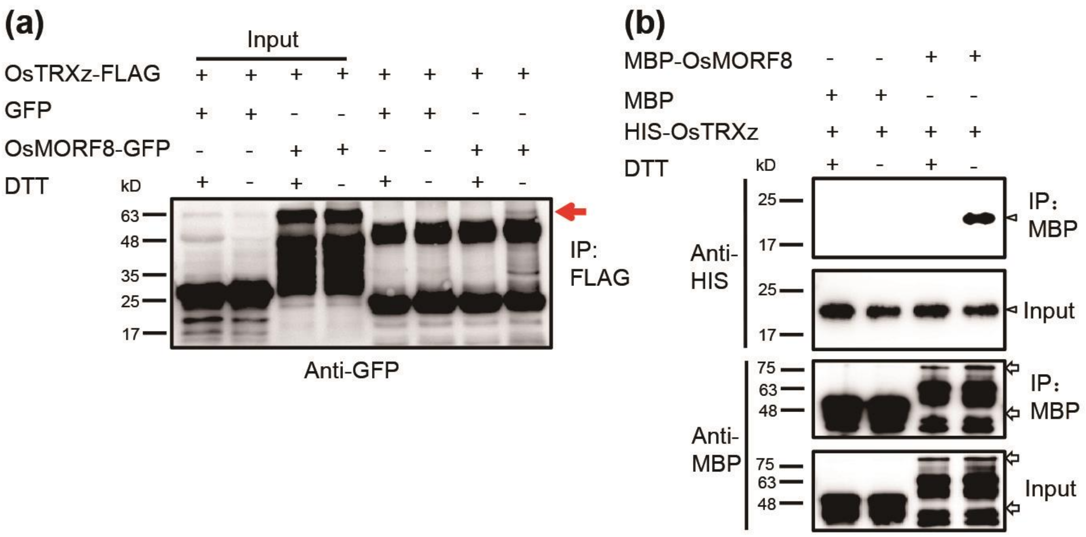

Fig. S9 DTT abolishes interaction between OsTRX z and OsMORF8. (a) *In vivo* Co-IP assays showing that only a minor portion of OsMORF8-GFP can be immunoprecipitated by anti-FLAG monoclonal antibody when OsTRX z-FLAG and OsMORF8-GFP are co-expressed in the leaf epidermal cells of *N.benthamiana***.** Addition of DTT disrupts the interaction. Red arrow indicates the precipitated band of OsMORF8-GFP. (b) *In vitro* pull-down assays of recombinant HIS-OsTRX z using resins containing MBP-OsMORF8. DTT treatment abolishes the interaction. Arrows indicate MBP and MBP-fusion proteins; Arrowheads indicate HIS-fusion protein.

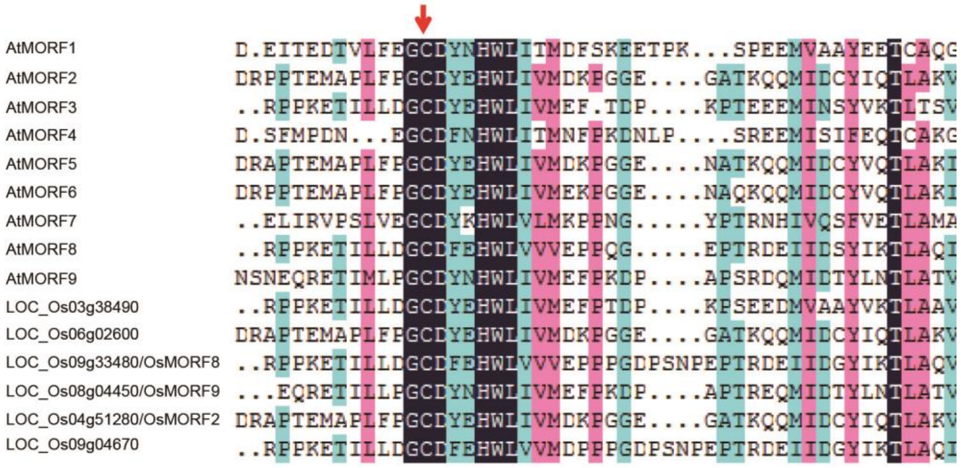

Fig. S10 Partial amino acid sequence alignment of all MORFs in rice and Arabidopsis. Red arrow indicates the conserved Cys residue.

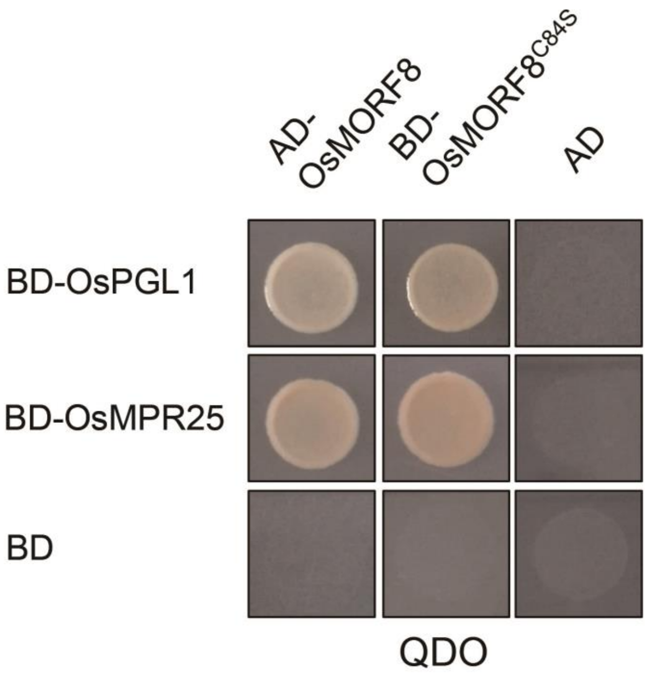

Fig. S11 Y2H assays showing the interactions between OsMORF8, OsMORF8^{C84S} and PLS-type PPRs. Y2H assays show that both OsMORF8 and OsMORF8^{C84S} interact with OsPGL1 and OsMPR25.

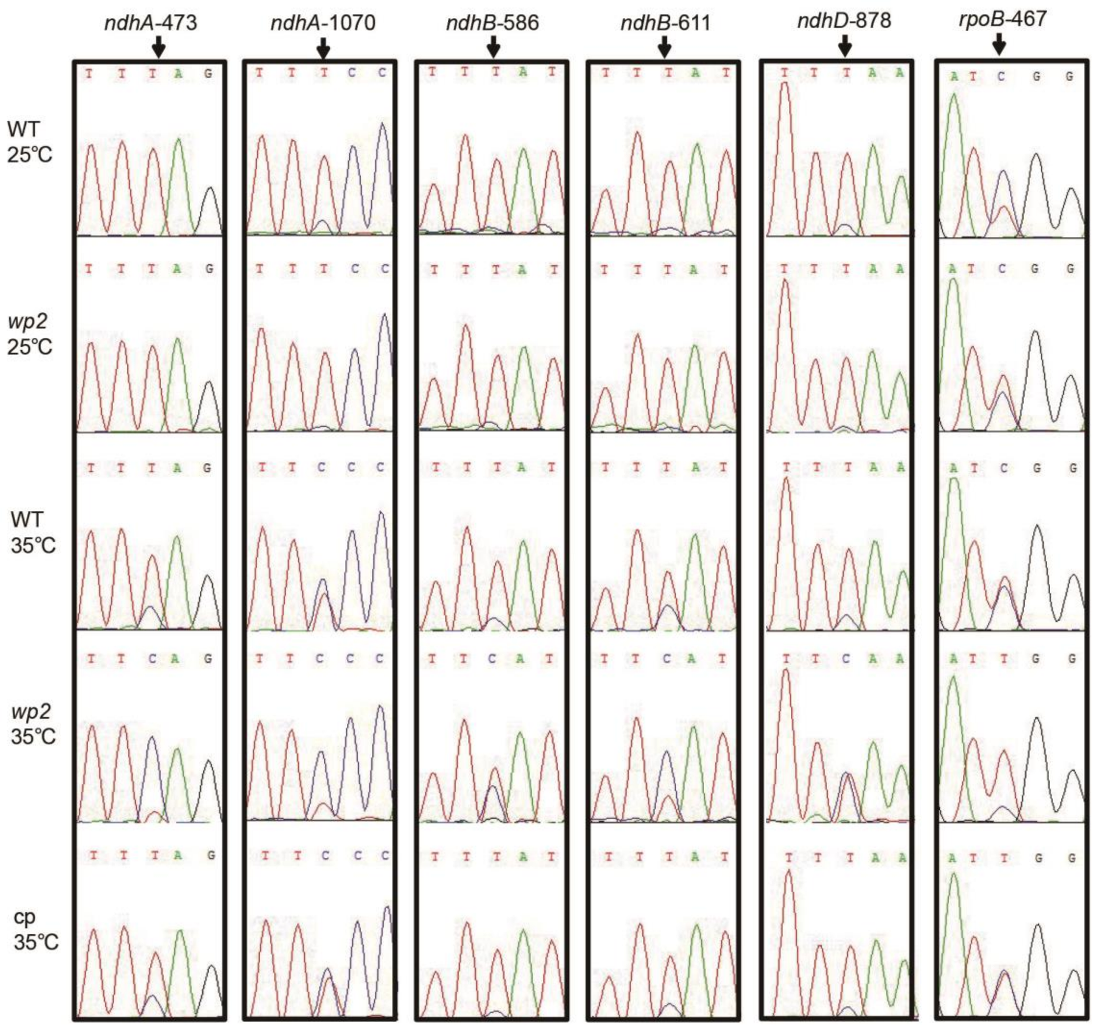

Fig. S12 Sequencing analyses showing the chloroplast RNA editing efficiencies of wild type, *wp2* mutant and complemented plants (cp) at 25°C and 35°C.

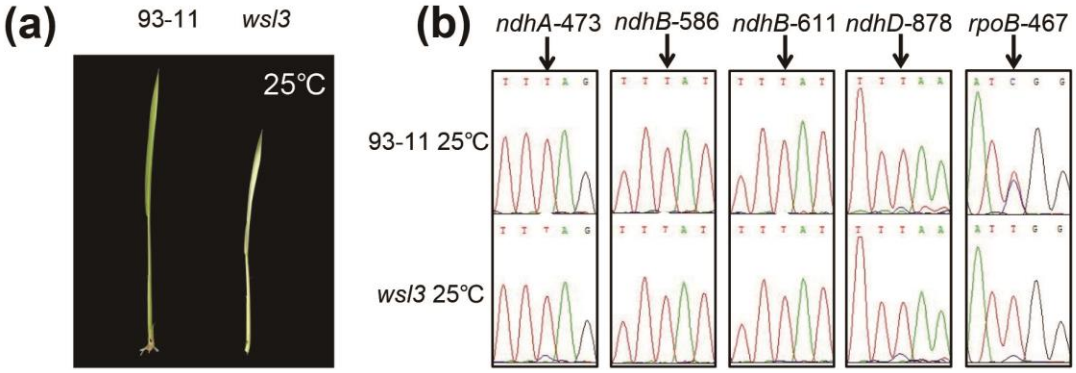

Fig. S13 Phenotype and chloroplast RNA editing levels of *wsl3*. (a) Phenotype of 10-d old seedlings of 93-11(wild type) and *wsl3* mutant grown at 25°C. (b) RNA editing efficiency analyses of several chloroplast editing sites in 93-11 and *wsl3* at 25°C.

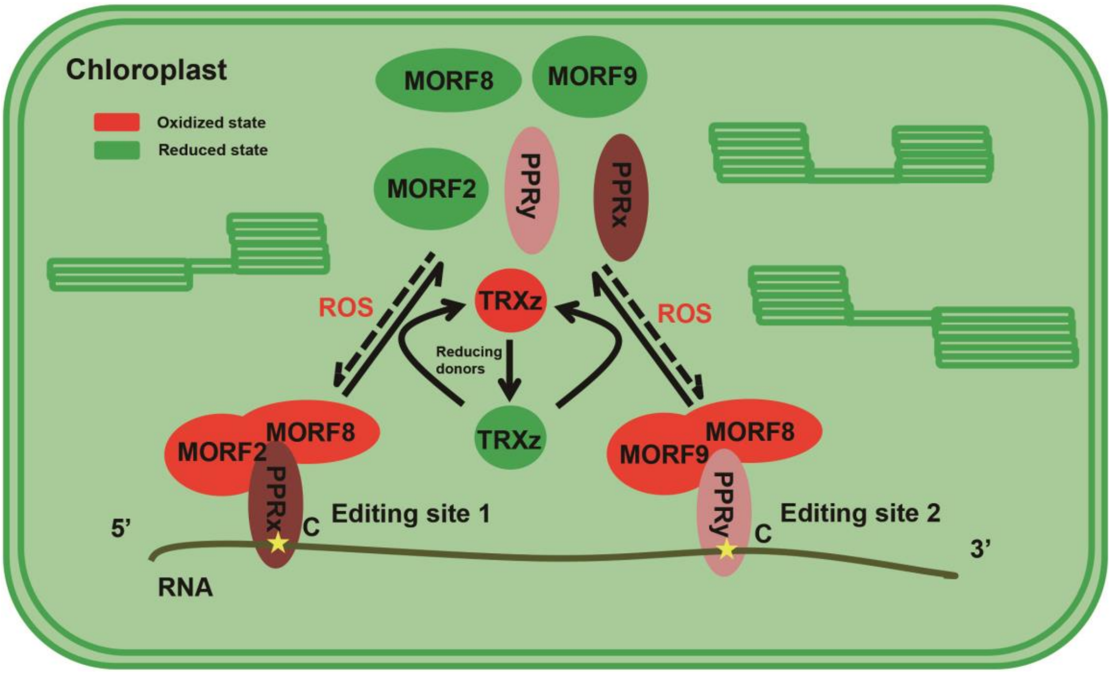

Fig. S14 Model of TRX z regulation of plastidial RNA editing in plants. Oxidized MORF proteins form homo- or heterodimers that interact with selected PPR proteins to form functional editosomes. Once the editosome complete the editing at one site, it is dissolved through reduction of MORFs by TRX z. Oxidized TRX z is reduced by its reducing donors in the chloroplast. The editing of RNA at another site would require reassembly of a new functional editosome by recruiting re-oxidized MORFs (by ROS in the chloroplasts) and another site-specific PPR protein(s).

Table S1 Analysis of all known chloroplast editing sites in wild type, *wp2*, L1 and cp at 25°C and 35°C. +/- values indicate ±SD (n= 4).

Editing sites	WT 25°C %	*wp2* 25°C %	WT 35°C %	*wp2* 35°C %	L1 25°C %	L1 35°C %	cp 25°C %	cp 35°C %
NDHA-473	100 ± 0	100 ± 0	77 ± 5	11 ± 1	26 ±3	13 ± 1	100 ± 0	75 ± 2
NDHA-563	100 ± 0	100 ± 0	78 ± 45	3 ± 3	74 ± 11	5 ± 4	100 ± 0	69 ± 13
NDHA-1070	88 ± 1	90 ± 5	41 ± 1	17 ± 2	53 ± 4	17 ± 1	97 ± 1	44 ± 3
NDHB-467	100 ± 0	100 ± 0	100 ± 0	87 ± 3	100 ± 0	77 ± 2	100 ± 0	100 ± 0
NDHB-586	100 ± 0	100 ± 0	81 ± 5	57 ± 2	100 ± 0	42 ± 2	100 ± 0	88 ± 1
NDHB-611	100 ± 0	100 ± 0	69 ± 3	26 ± 2	85 ± 3	17 ± 1	100 ± 0	77 ± 2
NDHB-704	100 ± 0	100 ± 0	100 ± 0	100 ± 0	100 ± 0	100 ± 0	100 ± 0	100 ± 0
NDHB-737	95 ± 1	96 ± 1	16 ± 3	10 ± 3	66 ± 2	8 ± 2	100 ± 0	30 ± 4
NDHB-830	100 ± 0	100 ± 0	100 ± 0.	79 ± 4	100 ± 0	69 ± 3	100 ± 0	100 ± 0
NDHB-836	100 ± 0	100 ± 0	100 ± 0	89 ± 2	100 ± 0	82 ± 6	100 ± 0	100 ± 0
NDHB-1481	94 ± 2	95 ± 1	92 ± 2	86 ± 1	94 ± 1	83 ± 1	100 ± 1	91 ± 1
NDHD-878	89 ± 3	90 ± 6	84 ± 1	47 ± 2	68 ± 1	40 ± 1	96 ± 1	84 ± 1
NDHF-62	74 ± 3	89 ± 3	78 ± 1	87 ± 1	86 ± 1	84 ± 2	90 ± 1	82 ± 2
NDHG--11	75 ± 3	83 ± 5	49 ± 1	35 ± 2	72 ± 2	35 ± 2	87 ± 2	54 ± 1
NDHG-347	86 ± 3	94 ± 2	72 ± 1	56 ± 1	66 ± 1	52 ± 2	96 ± 1	78 ± 2
RPOB-467	34 ± 3	60 ± 4	56 ±	83 ±3	90 ± 4	83 ± 4	58 ± 4	49 ± 1
RPOB-545	41 ± 3	62 ± 4	79 ± 2	91 ± 3	88 ± 1	91 ± 3	60 ± 4	73 ± 3
RPOB-560	40 ± 4	61 ± 2	78 ±3	88 ±3	86 ± 2	88 ± 2	57 ± 4	72 ± 3
RPL2-2	78 ± 4	82 ± 3	65 ± 1	68 ± 1	71 ± 2	67 ± 1	84 ± 1	57 ± 1
RPS8-182	90 ± 1	94 ± 3	77 ± 1	86 ± 1	94 ± 2	85 ± 4	100 ± 1	74 ± 2
ycf3-185	100 ± 0	100 ± 0	96 ± 1	73 ± 5	97 ± 2	69 ± 5	100 ± 0	95 ± 5
RPS14-80	87 ± 1	89 ± 2	35. ± 1	22 ± 1	31 ± 2	24 ± 6	87 ± 1	36 ± 2
ATPA-1148	95 ± 3	97 ± 1	85 ± 1	82 ± 1	76 ± 1	79 ± 1	97 ± 4	87 ± 2

Table S2 Analysis of all known Arabidopsis chloroplast editing sites in wild type and *trx z*. +/- values indicate ±SD (n= 4).

Editing sites	WT %	*trx z* %
accD-794	96 ± 1	22 ± 2
accD-1568	70 ± 1	15 ± 1
clpP-559	58 ± 3	36 ± 1.00
matK-706	96 ± 1	65 ± 1
ndhB-149	100 ± 0	100 ± 0
ndhB-467	100 ± 0	90 ± 1
ndhB-586	100 ± 0	89 ± 1
ndhB-726	16 ± 2	0 ± 0
ndhB-746	100 ± 0	100 ± 0
ndhB-830	100 ± 0	100 ± 0
ndhB-838	100 ± 0	77 ± 1
ndhB-872	100 ± 0	90 ± 1
ndhB-1255	100 ± 0	100 ± 0
ndhB-1481	100 ± 0	100 ± 0
ndhD-2	44 ± 1	20 ± 1
ndhD-383	100 ± 0	100 ± 0
ndhD-674	100 ± 0	100 ± 0
ndhD-878	76 ± 2	65 ± 1
ndhD-887	100 ± 0	100 ± 0
ndhF-290	96 ± 1	50 ± 1
ndhG-50	95 ± 1	72 ± 1
petL-5	58 ± 2	6 ± 1
psbE-214	100 ± 0	85 ± 1
psbF-77	100 ± 0	81 ± 0
psbZ-50	84 ± 1	48 ± 1
rpl23-89	86 ± 2	60 ± 3
rpoA-200	68 ± 2	47 ± 2
rpoB-338	87 ± 1	89 ± 2
rpoB-551	90 ± 1	95 ± 1
rpoB-2432	89 ± 1	90 ± 1
rpoC1-488	52 ± 3	70 ± 1
rpl12-i-58	35 ± 1	38 ± 1
rps14-80	75 ± 1	76 ± 2
rps14-149	75 ± 1	76 ± 0
ATPF-92	100 ± 0	85 ± 2

Table S3 Analysis of all known chloroplast editing sites in 93-11 and *wsl3* at 25°C. +/- values indicate ±SD (n= 4).

Editing sites	93-11 %	*wsl3* %
NDHA-473	100 ± 0	100 ± 0.00
NDHA-563	98± 2	85 ± 1
NDHA-1070	66 ± 1	77 ± 1
NDHB-467	100 ± 0.00	100 ± 0
NDHB-586	100 ± 0.00	100 ± 0
NDHB-611	100 ± 0.00	100 ± 0
NDHB-704	100 ± 0.00	100 ± 0
NDHB-737	100 ± 0.00	100 ± 0
NDHB-830	100 ± 0.00	100 ± 0
NDHB-836	100 ± 0.00	100 ± 0
NDHB-1481	91 ± 2	91 ± 1
NDHD-878	89 ± 1	87 ± 2
NDHF-62	80 ± 2	78 ± 3
NDHG--11	90 ± 1	89 ± 1
NDHG-347	80± 1	89 ± 1
RPOB-467	63 ± 5	88 ± 1
RPOB-545	50 ± 4	76 ± 2
RPOB-560	43± 4	79 ± 1
RPL2-2	60 ± 0	56 ± 1
RPS8-182	88 ± 2	90 ± 1
ycf3-185	74 ± 1	63 ± 1
RPS14-80	96 ± 4	80 ± 3
ATPA-1148	90 ± 1	82 ± 1

Table S4 Primers used in this study

**Fine mapping**	Forward sequence	Reverse sequence
In1	TCTACCTCATTTAGCCCTAT	CATCACATCAAAACTTTCCT
In3	GGGAAGAAACGCAGGATGTA	TCTTTTCTTCATCTCGCTCA
In4	TGATTCGGAGCTTCTGTTCTTC	CATGATTGTTCGGTACGATGTC
In5	CAATGCTCACCTCATCCCAC	GAAATGCCGTAAACACCAAC
In6	AGTTGACTTGATATGCGGGG	TGGACATGGTAGCCTCAGAA
**Arabidopsis PCR**		
LP1	CCAACTACGCGACAAGGTATC	
RP1	TTTTCCACACCTCAACACTCC	
LBa1	TGGTTCACGTAGTGGGCCATCG	
AtTRX z	GCAGTAGAGTATGAGAGCAATGCA	CATCTCGTTGTCAATGATATCGTG
ACT2	GCCATCCAAGCTGTTCTCTC	GCTCGTAGTCAACAGCAACAA
**qRT-PCR**		
OsTRX z	TGAAGGTGGATACTGATGATGAA	TCCTTAGGGCGTCTTTGCT
PsaA1	GGGAGGTGGCGAGTTAGTAG	AATGCGTGAATGTGATGGAC
PsaA2	TTATCTTCAACGAGCGGT	TATCTCCAGGTCCTATTGTT
PsbD1	CTGCTACTGCTGTTTTCT	GATGTTATGCTCTGCCTG
Rpoβ	GTGGGGAACTTGCTTTAGG	GCTTGTTGTATCCGTCTGA
Rpoβ'	CATAGATTAGGCATACAGGC	AATAGCGGGAGATAGGAG
NADH2	ATCACTGTAGGACTTGGGTT	TTTCCAGAAGAAGATGCC
NADH4	TCCTTATTGCTTATGCTGTC	CCGTATGCTCCCATCTTTA
ATPα	TCAAAAAGGGCAAGATGT	TTGTAATGTAGCAGGGGAAT
ATPβ	TTATTGGACCCGTGCTGG	TTGCTTACCGTCAGTGTCTCG
RbcL	CAACTGTTTGGACTGATG	GTTACCCACAATGGAAGT
